# GPR17: Molecular modeling and dynamics studies of the 3-D structure and purinergic ligand binding features in comparison with P2Y receptors

**DOI:** 10.1186/1471-2105-9-263

**Published:** 2008-06-04

**Authors:** Chiara Parravicini, Graziella Ranghino, Maria P Abbracchio, Piercarlo Fantucci

**Affiliations:** 1 Laboratory of Cellular and Molecular Pharmacology of Purinergic Transmission, Department of Pharmacological Sciences, University of Milan, Via Balzaretti 9, 20133 Milan, Italy; 2 Delos S.r.l., via Lurani 12, Bresso, 20091, Italy; 3 Department of Biotechnology and Biosciences, University of Milano-Bicocca, Piazza della Scienza 2, 20126 Milan, Italy

## Abstract

**Background:**

GPR17 is a G-protein-coupled receptor located at intermediate phylogenetic position between two distinct receptor families: the P2Y and CysLT receptors for extracellular nucleotides and cysteinyl-LTs, respectively. We previously showed that GPR17 can indeed respond to both classes of endogenous ligands and to synthetic compounds active at the above receptor families, thus representing the first fully characterized non-peptide "hybrid" GPCR. In a rat brain focal ischemia model, the selective *in vivo *knock down of GPR17 by anti-sense technology or P2Y/CysLT antagonists reduced progression of ischemic damage, thus highlighting GPR17 as a novel therapeutic target for stroke. Elucidation of the structure of GPR17 and of ligand binding mechanisms are the necessary steps to obtain selective and potent drugs for this new potential target. On this basis, a 3-D molecular model of GPR17 embedded in a solvated phospholipid bilayer and refined by molecular dynamics simulations has been the first aim of this study. To explore the binding mode of the "purinergic" component of the receptor, the endogenous agonist UDP and two P2Y receptor antagonists demonstrated to be active on GPR17 (MRS2179 and cangrelor) were then modeled on the receptor.

**Results:**

Molecular dynamics simulations suggest that GPR17 nucleotide binding pocket is similar to that described for the other P2Y receptors, although only one of the three basic residues that have been typically involved in ligand recognition is conserved (Arg255). The binding pocket is enclosed between the helical bundle and covered at the top by EL2. Driving interactions are H-bonds and salt bridges between the 6.55 and 6.52 residues and the phosphate moieties of the ligands. An "accessory" binding site in a region formed by the EL2, EL3 and the Nt was also found.

**Conclusion:**

Nucleotide binding to GPR17 occurs on the same receptor regions identified for already known P2Y receptors. Agonist/antagonist binding mode are similar, but not identical. An accessory external binding site could guide small ligands to the deeper principal binding site in a multi-step mechanism of activation. The nucleotide binding pocket appears to be unable to allocate the leukotrienic type ligands in the same effective way.

## Background

Adenine (ATP, ADP), uracil (UTP, uridine 5'-diphosphate, UDP) and sugar nucleotides (e.g., UDP-glucose and UDP-galactose) are universal and phylogenetically-ancient signaling molecules involved in a multitude of biological processes, from embryogenesis to adult homeostasis. Actions of extracellular nucleotides on target cells are mediated by specific membrane receptors: the ligand-gated P2X channels, and the G protein-coupled P2Y receptors, which are widely distributed in human tissues [1].

P2Y receptors have recently attracted a lot of interest from the scientific community, since they belong to the 7-transmembrane (TM) rhodopsin family of G-protein-coupled receptors (GPCRs), which are the target of more than 60% of currently marketed drugs [2]. Besides the already characterized GPCRs, the recent publication of the human genome has revealed the presence of more that 100 "orphan" GPCRs, i.e., receptors responding to yet-unidentified endogenous ligands. Due to the crucial roles of GPCRs in human pathophysiology, their "deorphanization" is believed to unveil novel biological targets for drug discovery. Of interest for the purinergic field, several orphan GPCRs are closely structurally and phylogenetically related to the P2Y receptor family (see also below).

Eight distinct P2Y receptors are currently recognized: the P2Y_1,2,4,6,11,12,13,14 _receptors [1]. The missing numbers in the P2Y_1–14 _sequence represent GPCRs cloned from nonmammalian vertebrates or receptors for which a functional response to nucleotides has not yet been convincingly demonstrated. Pharmacologically, P2Y receptors can be subdivided into (1) adenine nucleotide-preferring receptors mainly responding to ADP and ATP. This group includes human and rodent P2Y_1_, P2Y_12_, and P2Y_13_, and human P2Y_11_; (2) uracil nucleotide-preferring receptors. This group includes human P2Y_4 _and P2Y_6 _responding to either UTP or UDP; (3) receptors of mixed selectivity (human and rodent P2Y_2_, rodent P2Y_4 _and, possibly, P2Y_11_); and (4) receptors responding solely to the sugar nucleotides UDP-glucose and UDP-galactose (P2Y_14_) [1]. From a phylogenetic and structural (i.e., protein sequence) point of view, two distinct P2Y receptor subgroups characterized by a relatively high level of sequence divergence have been identified [1,3,4]. The first subgroup includes P2Y_1,2,4,6,11 _subtypes and the second subgroup encompasses the P2Y_12,13,14 _subtypes. Alignment of the deduced amino acid sequences of the cloned P2Y receptors has shown that the human members of this family are 21 to 48% identical. The highest degree of sequence identity is found among the second subgroup of P2Y_12,13,14_. Due to wide involvement in regulation of physiological phenomena, dysfunctions of nucleotides and their receptors have been associated to various human diseases, including immune and ischemic/inflammatory conditions (ibidem).

Cysteinyl-leukotrienes (cysteinyl-LTs, such as LTC_4_, LTD_4 _and LTE_4_) are inflammatory lipid mediators generated by 5-lipoxygenase metabolism of arachidonic acid acting through G protein-coupled CysLT_1 _and CysLT_2 _receptors and implicated in bronchial asthma, stroke and cardiovascular diseases [5].

Recent data highlight the existence of a functional cross-talk between the nucleotide and the cysteinyl-LT systems. Both types of mediators accumulate at sites of inflammation, and inflammatory cells often co-express both P2Y and CysLT receptors. In rat microglia, the brain immune cells involved in response to cerebral hypoxia and trauma, activation of P2Y_1 _and CysLT receptors mediates co-release of nucleotides and cysteinyl-LTs [6], which might, in turn, contribute to neuroinflammation and neurodegeneration. In human monocyte/macrophage-like cells, CysLT_1 _receptor function is regulated by extracellular nucleotides via heterologous desensitization [7], and, in the same cells, montelukast and pranlukast, two selective CysLT_1 _receptor antagonists [5], functionally interact with P2Y receptor signaling pathways [8]. Challenge of human mast cells with pro-inflammatory cytokine interleukin-4 induced a yet-unidentified elusive receptor responsive to both LTC_4 _and UDP [9]. Finally, there are close structural and phylogenetic relationships between the P2Y and CysLT receptor families. Both P2Y and CysLT receptors cluster together into the "purine receptor cluster" of GPCRs, which also includes a large number of "orphan" receptors still awaiting identification [10]. Among these receptors, Nonaka and co-workers identified GPR87 as the closest receptor to the P2Y_12,13,14 _subgroup [11]. These authors also identified four TM motifs which are fully conserved in both GPR87, P2Y_12,13,14, _CysLT_1 _and CysLT_2 _receptors and are not found in other GPCRs [11]. Based on these structural relatedness, they hypothesized that all these receptors should respond to both nucleotides and cysteinyl-LTs. However, while P2Y_12 _was found to be promiscuously activated by both nucleotides and CysLTE_4 _[11], GPR87 was subsequently reported to specifically respond to lysophosphatidic acid and not to be activated by either ATP, UDP or UDP-glucose [12]. This suggests that the presence of specific structural motifs may be necessary but not sufficient to unequivocally define the pharmacological specificity of a given receptor.

**Figure 1:**
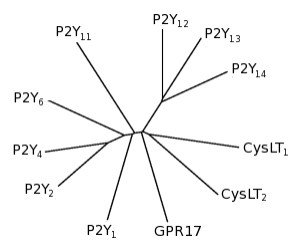
**Phylogenetic tree**. The cladogram shows the phylogenetic relationships between GPR17, P2Y and CysLT receptors.

Another member of the "purine receptor cluster" (GPR17), seemed particularly attractive to us, since it is located at intermediate phylogenetic position between P2Y and CysLT receptors and is the closest receptor to a common ancestor which also originated the P2Y_12,13,14 _and CysLT_1 _and CysLT_2 _(Figure 1).

On this basis, we have recently cloned and deorphanized GPR17; we demonstrated that its heterologous expression in a number of different cell lines results in the appearance of highly specific responses to both uracil nucleotides (e.g., UDP) and cysteinyl-LTs [13]. Agonists response profile of GPR17, as determined *in vitro *by [^35^S]GTPgammaS binding, was different from those of already known CysLT and P2Y receptors, with EC_50 _values in the nMolar and μMolar range, for cysteinyl-LTs and uracil nucleotides, respectively.

Several established P2Y and CysLT antagonists, namely, the P2Y_1 _selective antagonist 2'-deoxy-N6-methyladenosine 3',5'-biphosphate (MRS2179), the P2Y_12/13 _antagonist N(6)-(2-methyl-thioethyl)-2-(3,3,3-trifluoropropylthio)-beta, gamma-dichloromethylene-ATP (cangrelor), and the CysLT_1 _antagonists montelukast and pranlukast were found to be able to counteract GPR17 activation *in vitro *[13]. Both human and rat GPR17 are highly expressed in organs typically undergoing ischemic damage, i.e., brain, heart and kidney. Based on this and on the demonstration that both cysteinyl-LTs and nucleotides massively accumulate in ischemic brain [6,7] we also analyzed the role of GPR17 in a model of focal brain ischemia in the rat. *In vivo *inhibition of GPR17 achieved by either pharmacological agents able to counteract its *in vitro *activation (i.e., montelukast or cangrelor) or by the intracerebral injection of an anti-sense oligonucleotide specifically designed to knock down this receptor, dramatically reduced ischemic damage, suggesting GPR17 as the common molecular target mediating brain damage by nucleotides and cysteinyl-LTs. Thus, GPR17 is the first fully characterized "hybrid" GPCR responding to two unrelated families of non-peptide signalling molecules and represents a previously unexplored therapeutic target for brain ischemia.

The possibility of interfering with cerebral ischemia progression has obvious relevant implications for the development of innovative therapeutic approaches for management of human stroke. Based on the data summarized above, it can be anticipated that selective GPR17 antagonists may represent a novel class of neuroprotective agents able to counteract damage evolution [13]. Moreover, we anticipate that new chemical entities targeting both components of this dualistic receptor may prove extremely more effective than "standard" antagonists, thus leading to the development of novel dualistic pharmacological agents with previously unexplored therapeutic potential. However, none of the pharmacological agents utilized in the Ciana et al. study are really selective for GPR17, since montelukast is also active at CysLT_1 _receptors [5] and, conversely, cangrelor also inhibits P2Y_12 _and P2Y_13 _receptors [14,15]. On the other hand, the design and synthesis of selective GPR17 antagonist ligands would greatly benefit from the knowledge of receptor three-dimensional (3-D) structure and from the definition of its ligand binding mode. GPCRs are characterized by highly conserved structural topology, consisting of the seven TM helices bundle (TM1-7), the eighth amphipathic helix (H8), an extracellular N-terminus region (Nt), a cytoplasmic C-terminus tail (Ct) and three extracellular (ELs) and intracellular (ILs) loops connecting helices [16,17].

These structural features are shared among protein sequences that have very low similarity with the only 3-D structure so far known, i.e. bovine Rhodopsin (*b*Rh) [18,19].

Nevertheless, *b*Rh-based homology modeling combined with dynamic simulations and experimental data have been successfully used to investigate the ligand-receptor features of several GPCRs: this procedure has been demonstrated to be useful for rational drug design [20,21]. Since 1995, many studies have focused on ligand binding mode and on the design of selective nucleotide analogues for other nucleotide receptors, starting from P2Y_1 _[22-24]. Site-directed mutagenesis has been applied to the elucidation of P2Y receptor structure and ligand binding modalities. Some positively charged residues in TM 3, 6, and 7 of the P2Y_1 _and P2Y_2 _receptors have been shown to be crucial for receptor activation by nucleotides [25,26]. They probably interact with the negative charges of the phosphate groups of nucleotides, since it is known that the receptor ligands are nucleotidic species uncomplexed to magnesium or calcium. Actually, the eight P2Y receptors identified so far have a H-X-X-R/K motif in TM6. The P2Y_1_, P2Y_2_, P2Y_4_, P2Y_6_, and P2Y_11 _receptors share a Y-Q/K-X-X-R motif in TM7, whereas another motif, K-E-X-X-L is found in P2Y_12_, P2Y_13_, and P2Y_14 _receptors [1,4]. More recently, for P2Y_12_, P2Y_13_, and P2Y_14 _receptors, one additional lysine residue in EL2 has been suggested to be particularly important for nucleotide binding [27]. It would be interesting to assess if the same aminoacid residues proposed to be important for nucleotide binding in P2Y receptors are also involved in binding of GPR17 to purinergic ligands. The present work was specifically aimed at modeling the 3-D structure of GPR17, with the goal of designing new and selective ligands by defining the binding mode of its endogenous agonist UDP and of two nucleotide-derived compounds, such as MRS2179 and cangrelor, which have been reported to act as antagonists at this receptor (see above and Ciana et al., 2006).

## Results and Discussion

### The structure of the receptor

As a first step to the rational design of selective GPR17 ligands, a homology model of human GPR17 (*h*GPR17) was built using as a template the X-ray crystal structure of *b*Rh obtained at 2.20 Å resolution and deposited in the protein data bank as 1U19 (see also Methods) [19]. The sequence identity shared by *h*GPR17 and *b*Rh is only 21% (data not shown), that is the same order of magnitude shared by *b*Rh and other related nucleotide receptors for which modeling has been successfully applied for a long time. The sequence of GPR17 consists of 339 aminoacids, corresponding to the human receptor sequence in its shorter isoform [GPCRDB: Q13304-2].

Multiple alignment of GPR17 with P2Y receptors, CysLT receptors and *b*Rh, reported in Additional file: Figure 1 [see Additional file 1], showed the existence of two conserved cysteines among the various sequences (Cys104 and Cys181 in GPR17) which are conserved in the great majority of GPCRs. The corresponding cysteines in *b*Rh form a disulphide bridge; this structural feature was assumed also for GPR17.

**Figure 2:**
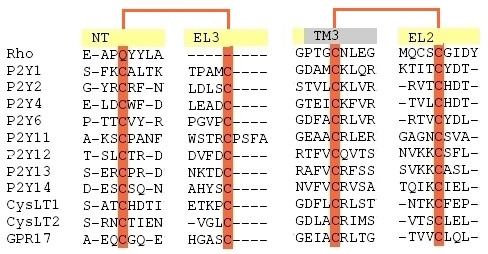
**Multiple sequences alignment**. The two pair of conserved cysteines discussed in the text are highlighted in red. A conserved disulphide bridge links Cys104 (EL2) and Cys181 (TM3); Cys23 (Nt) and Cys269 (EL3) form an additional disulphide bridge that seems to be a peculiar feature of a restricted subgroup of GPCRs among which GPR17, P2Y and CysLT receptors. See supplementary material for the full alignment.

The receptor has an additional pair of cysteines which are conserved in all the P2Y and CysLT receptors; these two residues are positioned at the end of the Nt (Cys23) and at the middle of the EL3 domain (Cys269), respectively (see also below). Interestingly, these residues are not present in *b*Rh. It has been demonstrated by site-directed mutagenesis and ligand affinity data that corresponding cysteines in P2Y_1 _form a disulphide bridge which is important for receptor activation [28]. Figure 2 shows a detail of the multiple sequence alignment of GPR17, *b*Rh, all the P2Y and CysLT receptors highlighting the formation of a second putative disulphide bridge. In agreement with our previous studies suggesting functional and phylogenetic relationships between GPR17, P2Y and CysLT receptors [13], we included this additional disulphide bridge into the 3-D model of GPR17.

The initial structure obtained from homology modeling was topologically close to the template; polar hydrogens were added and optimization of sidechains was run in cycles in which the backbone was kept fixed.

The locally minimised structure has been then embedded in a fully hydrated phospholipidic bilayer (dipalmitoyl-phosphatidyl-choline, DPPC, hydrated with water), as described in Methods.

The *b*Rh X-ray file derived from the crystal asymmetric unit reports 66 water molecules associated with both chain A and chain B. These molecules are localized in the vicinity of highly conserved residues and in the retinal pocket, and they are probably involved in the regulation of the activity of *b*Rh-like GPCRs [29].

In addition to the water molecules considered as explicit solvent, we have taken into account also all the solvent molecules from the pdb file free from stereochemical hindrances.

The first part of the molecular dynamics simulation is a simulated annealing (SA, see Methods for details concerning the warming-cooling cycles), in which the motion of helices is restrained; the root-mean-square difference (rmsd) for backbone atoms and sidechains between the initial model and the final structure is 2.80 Å.

Most of the water molecules that we included according to the X-ray data actually diffused into the solvent layer, with the exception of a few of them which remained inside the transmembrane bundle for the entire simulation time. These water molecules (labelled as Wat6808, Wat6809, Wat6812, Wat6813, Wat6814, Wat6815, Wat6816, Wat6817, Wat6822) were always close to the helical bundle due to the formation of favourable interactions with sidechains of the protein.

At the end of SA cycles, the mobility of some structural elements of the protein were considerably high, as shown by data in Table 1.

**Table 1:** Root mean square differences (rmsd) of structural regions of GPR17 after SA cycles

Structural domains	rmsd (Å)
Nt	5.9
EL1	4.8
EL	5.5
Ct	3.3
IL1	2.2
IL2	4.6
IL3	4.9

The structure of the protein-lipids-solvent system derived from the SA simulation was used as input for 10 ns of molecular dynamics (MD, NPT ensemble, T = 310 K, see Methods).

The stability of the molecular assembly was monitored by following the total energy of the system and by the rmsd of the C-α atoms trend as a function of time as shown in the Additional file: Figure 2 and Additional file: Figure 3 [see Additional file 1].

**Figure 3:**
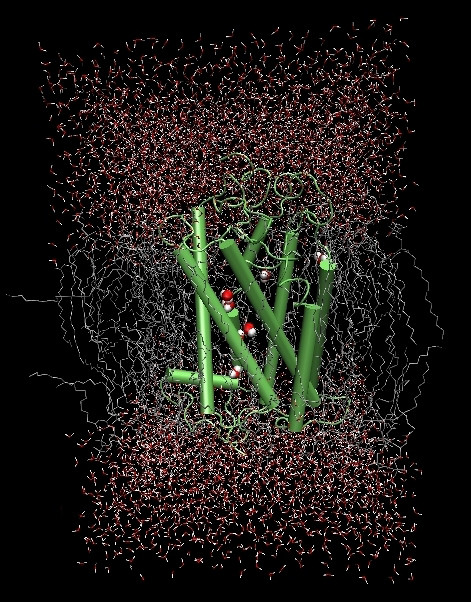
**Typical structure of GPR17 embedded in the fully hydrated lipid bilayer**. A frame of the system extracted from the10 ns MD simulations is shown. The backbone of the receptor is represented in green, the DPPC are in silver, water is in red/white and the internal water molecules are displayed as spheres.

The final picture of the protein after 10 ns of MD is shown in Figure 3 and in Additional file: Figure 4 [see Additional file 1]. The global structure of the protein remained quite similar to the initial one, although the structural domains were unrestrained.

**Figure 4:**
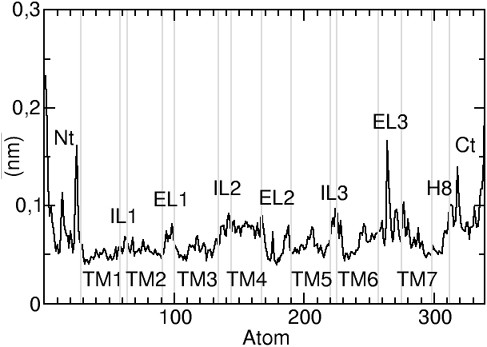
**Rms fluctuation of C-α atoms plotted as a function of the residue number**. The value of the fluctuations of the protein is not high in general, but some prominent peaks appear in the region of the Nt and EL3 domains.

As expected, the TM helical domains and the ELs and ILs regions showed markedly different dynamics behaviour, as reported in Figure 4, where the root mean square (rms) fluctuations are reported versus the residue number.

Among the loop regions, IL1, IL3 and EL1 were the most rigid, whereas EL2 moved towards the TM bundle and displayed a new network of contacts. As expected, the Nt and Ct regions were by far the most mobile regions. The arrangement of α-helices underwent little changes during the MD simulation, with the exception of TM7, which showed a mobility higher than other TM domains, as showed in Additional file: Figure 5 [see Additional file 1], but its rmsd value was never higher than 1 Å.

To ensure that the mobility of TM7 was not due to a loss of the α-helix structure but was indeed due to an intrinsic property of the protein domain, results were compared with those obtained with a "trial" run, performed by applying selective harmonic restraints to the interhelical H-bond distances of TM7 (see Methods for details). The need to apply local restraints to the α-helix backbone, in order to avoid a loss in secondary structure of TM7, has been previously found in other rhodopsin-based homology models of GPCR [30,31]. This probably arises because GPCR models are always obtained from the rhodopsin X-ray structure, where TM7 is stabilized by retinal, its bound ligand. However, in a recently published paper, Deflorian and co-workers reported that in their MD simulations of thyrotropin-releasing hormone receptor models (THR-R1 and THR-R2), no restraints were required to preserve the α-helical secondary structure of the TM segments [32].

**Figure 5:**
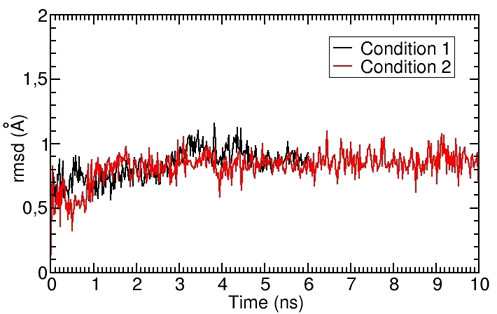
**Comparison of two different simulation methods in determining the mobility of TM7**. The plot shows the values of rmsd as a function of time obtained in two different simulation protocols, i.e., with NOE distance restraints (Condition 1, 6 ns, in black) or without NOE distance restraints (Condition 2, 10 ns, in red).

Comparison between MD simulations, performed with and without distance restraints, showed a mobility (rmsd of C-α atoms) similar for both simulations: near to 1.5 Å for the whole protein (Additional file: Figure 3) [see Additional file 1] and near to 1 Å for TM7 (Figure 5).

The number of the H-bonds observed during the MD simulations and the number of residues with α-helix geometry were comparable, as reported in Additional file: Figure 6 and Additional file: Figure 7, respectively [see Additional file 1]. This suggests that the observed mobility of TM7 does not impair either the α-helix topology or the global packing of the helical bundle. On this basis, and due to the proved stability of the trajectory, the subsequent runs were performed without the use of any "artificial" constraints but simply employing an explicit membrane environment closer to native conditions. Earlier studies have indeed shown that the mobility of α-helices embedded in membrane models is lower than the mobility of α-helices in water or methanol. For example, a simulation study of a TM Alamethicin helix in a palmitoyl-oleoyl-phosphatidyl-choline bilayer compared the conformational dynamics of the TM peptide with those of Alamethicin in either methanol or water. It was concluded that in either the bilayer or in methanol, there was little change from the initial helical conformation of the peptide C-α rmsd, while in water there were substantial changes of rmsd accompanied by a loss in α-helix structure for some regions [33]. For further information about the mobility and topology of GPR17, see Additional file Figure: 3, Additional file: Figure 4 and Additional file: Figure 5 [see Additional file 1].

The architecture of the helical bundle and the organization of the most interesting helices is described in the following subchapter.

### Interhelical interactions

The main intermolecular contacts formed during the MD run and likely contributing to receptor function are described in detail in Table 2 and compared with those assumed to be relevant for the "parent" receptor *b*Rh and for related purinergic receptors [27,30,23,34,35] (Table 3). For Table 3, the Ballesteros and Weinsten numbering system has been adopted [36]. For time evolution plots, see Additional file: Figure 8 and Additional file: Figure 9 [see Additional file 1]. We report below some of the most interesting observations emerged from this analysis.

**Table 2:** Residues involved in main functional interhelical interactions in GPR17

	TM2	TM3	TM4	TM5	TM6	TM7
TM1	D41-R87* D41-D77* N49-D77					Y38-S287 N310-R58 E30-R280 D41-S287 D41-N289* N49-N289 N49-Y297* N49-G290

TM2		T107-T86 D77-N114	H72-W156			R87-S287 D77-Y297* D77-N289 D77-D293 D77-G290 N67-K303*

TM3		D128-R129	T123-T208 Y116-A162	S126-Y212 T123-T208 Y116-S196	Y112-H252	S118-N289

TM6						C247-T286 Y251-T286

TM7						D293-N289 Y297-K303*

Main inter-helical networks			Residues involved in H-bonds/ionic interactions			
TM1-TM2-TM7			N49-D77-G290			
TM1-TM2-TM3			N49-D77-N114			
TM1-TM2-TM7			N49-D77-N289			

*Interactions involve water molecules.

**Table 3:** Comparison of functionally important motifs/residues conserved in GPR17 and related receptors

GPR17	GPCR (type/family)	SHARED FEATURES (1)
(residue number and structural domain)		
N49-D77-G290 TM1-TM2-TM7	*b*Rh	1.50–2.50–7.46
Y38-S287 TM1-TM7	P2Y	1.39–7.43
N114-D293 TM3-TM7	P2Y	3.35–7.49
H72-TRP156 TM2-TM4	*b*Rh	2.45–4.50
D128-R129 conserved interaction TM3-TM6 not conserved (3.50–6.30)	*b*Rh R of the DRY motif interacts with acidic residue in 6.30 (maintains the ground state) GPR17, P2Y, CysLT receptors have a basic residue in 6.30	D/E-R-Y/W motif 3.49–3.50–3.51
A233-M236	*b*Rh	Hydrophobic pocket accommodating DRY motif 2.33–2.36
H252-X-X-R255 TM6	P2Y, CysLT receptors in P2Y agonists mediate receptor activation/coordination of the phosphate moiety	H-X-X-R motif 6.52-X-X-6.55
N77-N289-D293 D2.50-N7.45-D7.49 TM2-TM7	*b*Rh	D2.50-N7.45-D7.49 TM7 residues belong to N/D-P-X-X-Y motif
G10-L17 V173-L182	*b*Rh	Plug β-hairpin in Nt and EL2
S118-water	*b*Rh	3.39-water
C104-C181	All GPCR	disulphide bridge TM3-EL2
C23-C269	P2Y, CysLT receptors	disulphide bridge Nt-EL3

(1)See Ballestero and Weinstein's numbering system for residue index [36].

The spanning of TM3 across the helical bundle seemed to divide the receptor in two well distinct regions characterized by different features. A first hydrophilic region encompassing TM1, TM2 and TM7 contained all the water molecules derived from crystallized *b*Rh. As in *b*Rh, starting from Arg87 and proceeding along the whole length of the protein, multiple hydrogen/ionic interactions between TM1, TM2, TM3 and TM7 stabilized the helix pack. Arg87, Asp41, Ser287, Asn289, Asp293, Asn114, Asp77, Asn49, Ser118, Tyr297, Lys303, Glu330, Asn67, Lys327 and five water molecules (Wat6817, Wat6815, Wat6816, Wat6809 and Wat6812) contributed to the formation of the internal polar network (Table 2). In addition, Ser118 could also interact with either Wat6814 or Wat6816, thus participating to the continuous H-bond network described above (Table 3). This residue (position 3.39) is conserved as a OH-bearing aminoacid in many GPCRs, including P2Y and CysLT receptors. In *b*Rh, this position is occupied by alanine and the OH group is provided by a water molecule involved, together with a sodium ion, in receptor activation [29] (Table 3). A second hydrophobic region, where aromatic residues are predominant, encompassed TM4, TM5 and TM6. Here, the aromatic residues Tyr112, Tyr116, Tyr120, Tyr251, Phe203, Phe203 and the highly conserved sub-pocket formed by Phe201 (5.47), Phe244 (6.44) and Phe248 (6.48) constituted an aromatic cluster between TM3, TM5 and TM6. In TM3, three subsequent tyrosine residues (Tyr112, Tyr116 and Tyr120) faced the hydrophobic cavity delimited by TM5 and TM6. The first two residues are conserved in most P2Y and CysLT receptors, but not in *b*Rh, and they are probably involved in stabilization of the interhelical interactions, as suggested by our dynamics simulation.

The outmost part of TM3 seemed to be permanently engaged in a conserved disulphide bridge with EL2 involving the Cys104 and Cys181 residues, which is an essential structural constraint for most GPCRs, as already mentioned above [18] (Table 3). This disulphide bridge constrained the whole structural organization of the protein. The bending of EL2 caused the formation of a plug that shields the extracellular side of the protein from the transmembrane space. In *b*Rh, as in many other GPCRs, this plug seems to prevent the outing of embedded ligands. In P2Y_1_, the role of this disulphide bridge has been further investigated through mutagenesis data confirming its importance for receptor trafficking to the membrane [28].

Within TM3, a ionic binding is likely to occur between Asp128 and Arg129. These two charged aminoacids are positioned at the intracellular end of TM3, and belong to the highly conserved D(E)-R-Y(W) motif (Table 3). In *b*Rh, the corresponding salt bridge (Glu134-Arg135), together with the interaction between Arg135 (3.50) and Glu247 (6.30) is believed to keep the receptor in its inactive state [37]. Alignment of GPR17 with *b*Rh, did not reveal any corresponding acidic residue in the TM6 of GPR17. In analogy with P2Y and CysLT receptors, at position 3.50, GPR17 displays a basic residue instead of an acidic one (Table 3). However, we observed that, during the simulations, Arg129 can form a stable ionic binding with the Glu330 belonging to the Ct of the protein.

As in *b*Rh, in GPR17, a hydrophobic pocket formed by Ala233 and Met236 accommodates the Asp128-Arg129 ionic couple (indicated as D128-R129 in Table 3) of the D-R-Y motif [38].

An additional H-bond between Arg3.50 and a generic H-bond acceptor at position 6.34 have been proposed for all members of the P2Y_12_-like subfamily [27]. In GPR17, as in the P2Y_1 _subgroup of receptors, this position is occupied by a hydrophobic residue.

TM6 contains the H-X-X-R/K motif typical of all P2Y receptors and conserved among few related receptors, including CysLT receptors (Table 3). Experimental data demonstrates that, in P2Y_1_, both histidine (His277) and lysine (Lys280) are essential for ligand recognition and/or receptor activation [3,25]. In particular, Lys280 (6.55) coordinates the phosphate moiety of nucleotide ligands, while His277 (6.52) is probably implicated in agonist-mediated receptor activation [26,31,39]. In our GPR17 model, these two crucial residues are His252 and Arg255. The first engaged polar contacts with residues from EL2; the second is the best candidate residue for nucleotide binding. Experimental data from mutagenesis studies will help confirming this hypothesis.

Our MD simulation also showed that TM6 engaged only few interactions with other helices (the same was observed for TM4, that is characterized by a high content in hydrophobic residues). However, as outlined above, TM6 contains the putative critical motifs for binding, suggesting that this helix may maintain a dynamic behaviour needed to evoke receptor activation without constraints from the other helices. In fact, in *b*Rh, TM6 is believed to move away from TM3 thereby starting the activation process [40].

### Intracellular regions

The abundance of hydrophilic and charged residues of the intracellular domains results in the formation of a complex weave of polar interactions: this is not discussed in detail here, due to its minor relevance to the purpose of the present study.

### Extracellular regions

Despite the length and the flexibility of the Nt, we observed a pronounced structural stabilization after an initial significant conformational change. This resulted in the formation of a typical β-hairpin running nearly parallel to the horizontal plane of the membrane. This secondary structure faced a second beta strand present in EL2, but oriented in the opposite direction, forming a plug that is commonly believed to be critical for receptor activation mechanism [41]. The relative position of these two β-hairpins was strongly influenced by the presence of the disulphide bridge. Despite the relatively low sequence identity between *b*Rh and GPR17, this typical organization of the EL2 and Nt regions appeared to be conserved.

As for the helical domains, we report below some of the intramolecular interactions observed in the extracellular part of the protein during our MD simulation.

Residues from Gly10 to Leu17 in Nt and from Val173 to Leu182 in EL2 were involved in the formation of two slightly distorted β-hairpins. The sidechain of Gln174 in EL2 pointed toward the Nt, forming a H-bond with the backbone of Leu11 in Nt, and directly connecting the two β-strands. Furthermore, the backbone of Gln174 and Asn176 were in H-bond contact with Gln22 in the first β-hairpin.

Glu21 in Nt was bound to Asn31 and Asn95 belonging to TM1 and TM2, respectively. Thr13 in Nt was bound to Ser196 and Arg105 of TM3, which, in turn, interacted with the peptide carbonyl of several residues located close to the extracellular end of TM4. Asn14 and Ser16 of the β-hairpin were bound to Ser196 and Glu103, respectively. Gln183 pointed towards the interhelical space and appeared to be involved in a H-bond network running all along the helical bundle.

All the interactions reported above, together with other intra-chains interactions, form a compact, highly structured, extracellular plug encompassing both EL2, EL3 and Nt. This region is believed to restrain conformational changes for the resting state and control binding mechanisms during receptor activation.

All these structural evidences suggest that, in GPR17, the region formed by the EL2, EL3 and the Nt would play a critical role in receptor activation and ligand recognition, at least as an "accessory" pocket, favouring the access of small ligands to the deeper principal binding site (see below), in a multi-step mechanism of activation. In this context, EL1 appears to play a minor role because of its limited length and predominant hydrophobic nature.

Involvement of extracellular domains in nucleotide recognition has been suggested for the first time by Moro and co-workers for P2Y_1_. These authors proposed the existence of two meta-binding sites and a path of access of the ligand to the principal intracellular binding sites [42]. Furthermore, in P2Y_1_, some charged residues believed to be critical for receptor function in EL2 and EL3 have been successfully probed throughout mutagenesis combined with ligand affinity measurements [26,27,34]. These experiments confirmed the above hypothesis and, at the same time, support our finding and conclusions.

### Definition of the binding site

A general configuration of the binding sites for all known P2Y receptors was proposed based on docking and mutagenesis studies [27,35]. It is commonly assumed that, in these receptors, the phosphate moiety of nucleotide ligands can be accommodated in a positively charged pocket formed by three residues. It has been also proposed that the nucleotide binding mode is specific and slightly different between the two subgroups of the family. For P2Y_1_,_2_,_4_,_6_,_11_, residues surrounding the phosphate chain are all located in transmembrane domains and correspond to 3.29, 7.39 and 6.55. In the case of P2Y_12_,_13_,_14_, two of these three residues (6.55 and 7.35) belong to TM6 and TM7, respectively; the third one is a lysine which is located in EL2, in the vicinity of the conserved cysteine [27]. At variance from this model, binding of UDP-glucose to P2Y_14 _has been recently reported to be quite different from that of UDP to P2Y_6 _[31]. Indeed, two basic sidechains found essential for the agonist binding site in P2Y_6 _and all previously known P2Y receptors were not involved in P2Y_14 _and are absent in the GPR17 sequence.

Multiple alignment with P2Y family members shows that GPR17 possesses only one of these three basic residues, in particular, residue 6.55 corresponding to Arg255 and belonging to the H-X-X-R motif typical of all P2Y receptors. Residues 3.29 and 7.39 correspond to Gly108 and Ser283, respectively; the first residue cannot display sidechain interactions, but is able to enhance the flexibility of the chain. The role of the EL2 has been also investigated in several P2Y receptors. The lysine which is present in the EL2 in P2Y_12_,_13_,_14 _is not conserved in GPR17, but, interestingly, is conserved in CysLT_1_.

It has been also proposed that an acidic residue, located two positions ahead of the conserved cysteine, would play an important role in ligand recognition. This critical residue is aspartic acid in P2Y_1_,_2_,_4 _and corresponds to Glu174 in P2Y_14_, a receptor where an additional glutamic acid on EL2 (Glu166) seems to participate to the stabilization of the ligand-receptor complex.

GPR17 lacks the charged residues close to this conserved cysteine: the nearest ones (Arg186 and Glu187) are shifted toward the Ct end of EL2 in the direction of TM5. Interestingly, glutamic acid in EL2 is conserved as it is in CysLT receptors.

Due to the relatively low identity between GPR17 and related receptors sharing both endogenous and synthetic ligands, sequence analysis does not provide an exact definition of ligand binding mode, despite the increasing knowledge on the arrangement of nucleotides in ligand-receptor complexes of P2Y receptors. The characterization of the binding site of cysteinyl-LTs are even more ill defined.

Mutagenesis studies on P2Y_1 _receptor suggest that residues 3.29 and Asp204 (EL2) are involved in the receptor activation caused by ligand binding [26,28]. The role of this pair of charged aminoacids has been postulated also for P2Y_6_, where residue 3.29 is involved in an ionic interaction with Glu179 (in EL2) and this is crucial for the retention of receptor ground state conformation. The phosphate interaction with the sidechain of Arg103 is supposed to destabilize the ion pair Arg103-Glu179 and this mechanism could take part in receptor activation [30].

Our GPR17 model indicates a different and specific distribution of charged residues in the area corresponding to the nucleotide binding pocket of P2Y receptors suggesting that ligand-mediated activation may be different from that described for P2Y_6 _(see also below). In GPR17, ligand recognition (and receptor activation) is not mediated by the interplay of a ionic couple stabilizing the reciprocal EL2 and TM3 positions. The positively charged residue nearest to 3.29 in TM3 of GPR17 is Arg105, which is conserved as a positively charged residue also in all P2Y receptors, except for P2Y_12_. In our model, Arg105 was likely to form stable interactions with Tyr13, Tyr172 and Ser196 sidechains.

Conversely, in GPR17, the putative negatively charged counterpart of the 3.29 residue corresponds to Gln183, which was likely to form persistent H-bonds with Tyr116, Ser196 and His192.

As a conclusion, detailed architecture of interactions between TM3, TM5, TM6 and EL2 showed that, in GPR17, a different and specific distribution of residues could stabilize the position of EL2 over TM domains, thus suggesting a slightly different mechanism of receptor activation (see also below).

### The bound agonist and antagonist ligands

The overall picture of the agonist-receptor complex was obtained by means of MD runs of 3 ns starting from the best-docked configuration of one of the natural ligands of GPR17, i.e. UDP.

In the 3-D arrangement of 7TM receptors, the binding pocket that Delos [43] (see Methods for more details) spotted as the most probable one which best accommodates the agonist ligand corresponded to the already well documented "nucleotide binding site" [35]; as expected on the basis of the poor conservation of sequences, the interatomic connections were different.

**Figure 6:**
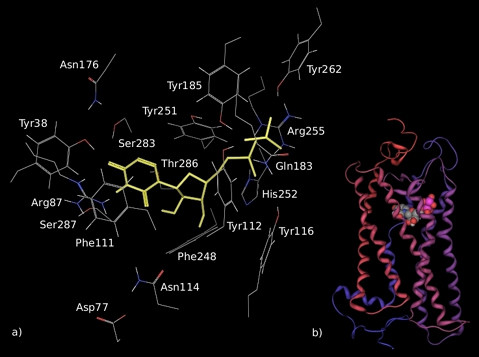
**Model of the complex formed by UDP and GPR17 after 3 ns of MD simulation**. UDP is displayed in yellow within the binding pocket in the detailed picture (a) and as spheres in the schematic representation of the entire ligand-receptor complex (b). In the tube representation, helices are coloured with a spectrum of colours whose gradient ranges from red (TM1) to blue (TM7). Spheres are coloured by element type: carbon is in grey, oxygen is in red, nitrogen is in blue, phosphorous is in purple and polar hydrogen is in white.

The putative arrangement of the UDP molecule is displayed in Figure 6. A multitude of possible interactions held the diphosphate moiety in place. The guanidine group of Arg255 (6.55) seemed to form an ionic bond with both the α and β-phosphates, with a shorter distance to the α-phosphate. Furthermore, the β-phosphate seemed to form a network of H-bonds with the hydroxyl groups of Tyr185 (EL2), Tyr112 (TM3) and Tyr262 (EL3) and to interact with the sidechain (N1) of His252 (TM6). Gln183 (EL2) was bound to both the α-phosphate and β-phosphates: as a consequence, Gln183 did not seem to coordinate the three tyrosines as in the unbound receptor.

In addition to the directly bound Gln183, another residue belonging to EL2, namely Gln171, pointed toward the intracellular space and kept its sidechain close to the phosphate chain in the pocket. Sidechains of His192 (TM5), Tyr116 (TM3), Ser196 (TM5), Tyr251 (TM6) were also within the binding pocket. The most important interaction of the agonist phosphates seemed to occur with Arg255; however, interaction with His252 (whose total charge was in our case assumed to zero) could be as important.

On the opposite side of the helical bundle, the uridine ring was coordinated by residues from TM7 and TM1. Namely, the 4-O of uracil was likely to interact with the hydroxyl groups of Ser283 (TM7) and Tyr38 (TM1), whereas its 3-NH group seemed to interact with Ser287 (TM7). These polar interactions were the only ones present in the aromatic subsite hosting the uridine ring. Mostly hydrophobic contacts were instead observed within the pocket: these involved Tyr251, Phe111, Phe248, Tyr112. An additional intramolecular H-bond was formed between Asn176 in EL2 and Ser283 in TM7, involving the OH group of serine and NH_2 _group of asparagine.

Although holding some peculiar features, the overall configuration of the agonist ligand agreed with the general configuration predicted in previous computational papers for P2Y receptors, including the P2Y_6 _receptor that shares the common agonist UDP with GPR17 [30]. Analogies concern the involvement of the conserved residues Ser283 (7.43), Tyr38 (1.39) and Phe111 (3.32) in the coordination of the nucleobase and the electrostatic interaction between the conserved Arg255 (6.55) and the phosphate moiety. Ser7.43 was also involved in the binding of the uracil group of UTP in P2Y_2 _and P2Y_4_, in the binding of the adenine group of MRS2179 (see also below), and, in general, of the nucleobase in P2Y receptors. The same residue has also been described as essential for the activation of P2Y_1 _[35].

In our model, the sugar moiety of UDP established only a few specific interactions with 7TM regions. The most important interaction engaged by the ribose involved the 2'-OH group of the sugar, that, during the MD simulation, shifted from Asn114 (3.55) to Thr286 (7.42); conversely, the 3'-OH pointed to TM6, but was not directly involved in any specific H-bond. In the most representative UDP structures of the whole MD simulation, the ribose clustered in a Southern (S) conformation, while the starting configuration was Northern (N). The shift from (N) to (S) conformation seemed to proceed in parallel to the shift of the 2'-OH group of the agonist ligand from TM3 to TM7. This allowed the ribose to assume the conformation required to hold the phosphate and the uracil groups in the proper position for a stable receptor binding. Although, for GPR17, further experimental investigations are necessary to confirm this issue, similar data were reported for the ribose group of UDP-glucose in binding to P2Y_14_. For this receptor, the 2'-OH group was bound to Asn3.35 in the (N) ribose conformation or to Asn7.45 and Ser7.42 in the (S) conformation, while the 3'-OH group never interacted with the receptor [31]. In the case of P2Y_1,2,4,11 _receptors, the (N) conformation of the pseudo-rotational cycle of the sugar enhanced the binding of adenine and uracil agonists [44]. In the case of P2Y_6_, the ribose group of UDP established specific interactions with the TM residues and this was related to the stabilization of the final active (S) conformation of the ribose. This unique profile of UDP binding in P2Y_6 _differs from the typical (N) conformation of uracil (and adenine) nucleotides in other P2Y receptors.

We then modeled two adenine nucleotide P2Y receptor antagonists that have been previously shown to interact with GPR17 [13]: MRS2179 and cangrelor.

**Figure 7:**
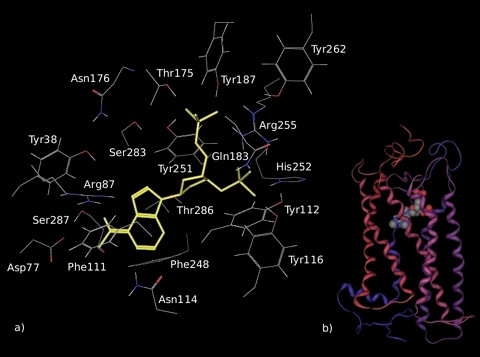
**Model of the complex formed by MRS2179 and GPR17 after 3 ns of MD simulation**. MRS2179 is displayed in yellow within the binding pocket in the detailed picture (a) and as spheres in the schematic representation of the entire ligand-receptor complex (b). In the representation of the whole receptor-ligand complex, helices and spheres are coloured as indicated in Figure 6.

The ribose-modified nucleotide analogue MRS2179, a selective P2Y_1 _receptor antagonist, binds to the same binding pocket of endogenous ligands on this receptor. In analogy with P2Y_1_, our docking experiments suggested that MRS2179 and UDP occupy a common region also on GPR17. The final picture of the bound antagonist is reported in Figure 7. The phosphate moiety was anchored to the same Arg255 residue that bounded to UDP by electrostatic interaction, but in a middle position between the two phosphate chains, as previously described for binding of MRS2179 to P2Y_1 _[35]. In addition, both the 3' and 5' phosphate chains were stabilized via H-bonds with many other residues in the binding pocket. The 3'-phosphate group formed a H-bond with the sidechain (N1) of His252 (TM6); the OH group of Tyr116 (TM3) interacted with the same oxygen atom of the 3'-phosphate group. The NH_2 _group of Gln183 (EL2) was H-bounded to the 3'-phosphate of MRS2179. The 5'-phosphate group was bound to the OH groups of Tyr185 (EL2), Thr175 (EL2) and Tyr251 (TM6). It is noticeable that, in the case of the antagonist, two solvent molecules (Wat6789 and Wat6808) were bridging the interaction of the phosphate groups with Asn279 (TM7) and Tyr262 (EL3), respectively. The adenine group of MRS2179 is likely to offer several nitrogen acceptor atoms to residues able to form H-bonds: in our model, the N7 was connected to Arg87 (TM2), the N6 interacted with Ser7.43 (TM7), the N1 interacted with Asn114 (TM3) and, finally, the N3 interacted with the backbone of Phe111. Here again, the hydrophobic residues Phe111 and Phe248 accommodated the nucleobase.

**Figure 8:**
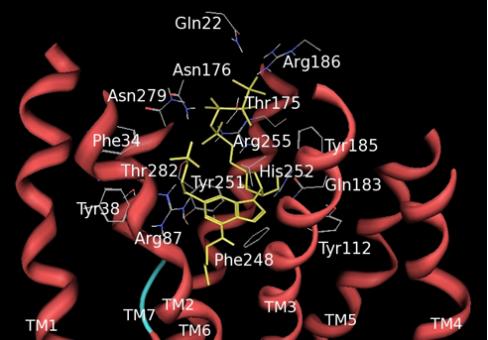
**Model of the complex formed by cangrelor and GPR17 after 6 ns of MD simulation**. Cangrelor is displayed in yellow embedded in the putative binding site; helices are coloured in red.

Cangrelor (previously known as ARC-69931MX [1]) is a potential anti-thrombotic agent due to its ability to counteract ADP-induced activation of platelet P2Y_12 _receptors responsible for platelet aggregation. Cangrelor is also a potent antagonist at the P2Y_13 _receptor subtype [15] and, as recently reported by us, at human and rodent GPR17 [1]. Its ability to potently prevent the progression of ischemic injury in a rat model of focal brain ischemia [13] also highlights this compound as a potential anti-stroke agent. Since no data on the binding mode of this antagonist at either P2Y_12 _or P2Y_13 _receptors are available, we decided to investigate its interaction with GPR17 by means of MD simulations. The configuration of the GPR17-cangrelor complex docked in the nucleotide binding pocket after 6 ns of MD is shown in Figure 8.

As in the case of UDP and MRS2179, a main driving interaction involved the phosphate groups (in particular the α-phosphate) and the basic Arg255 residue. Cangrelor accommodated the triphosphate chain between helices TM3, TM5, TM6 and TM7 and pointed to the extracellular space extending towards the EL2 residues. Due to the length of the phosphate containing chain, cangrelor conformation was more influenced by residues in extracellular loops with respect to smaller ligands, such as MRS2179 and UDP. An additional basic residue (Arg186) from EL2 was bound to the phosphate chain. Furthermore, four polar residues from EL2 (Asn176 and Thr175) and TM7 (Asn279, Thr282) directed their sidechains towards the transmembrane region and contributed to the stabilization of the complex by forming H-bonds with the phosphate chain.

Due to the anchoring of its phosphate groups from the extracellular side, cangrelor adenine moiety occupied a region between TM3 and TM7, but its orientation in the pocket differed from that of MRS2179. The amino group pointed towards the extracellular side as a result, which has never been proposed for adenine nucleotides docked at the P2Y receptors (see also below). In detail, the pyrimidinic ring faced TM1, TM2 and TM7, allowing the N1 and N6 nitrogen atoms of the ring to form H-bond interactions with Tyr38 and Arg87, while the imidazole portion of the molecule was directed towards TM3, TM5 and TM6 and was H-bound to the Gln183 sidechain of EL2. The adenine ring substituents, i.e., the 3,3,3-trifluoropropylsulfanyl and the 2-methylsulfanylethylamino chains elongated to a deeper region within the helical bundle and the extracellular side of the protein, respectively, thus extending to a receptor region which is inaccessible to small nucleotides such as UDP and MRS2179. Finally, the ribose moiety was positioned between TM3, TM5, and TM6, was surrounded by Tyr112 (TM3), Tyr116 (TM3), His252 (TM6), Arg255 (TM6), Gln183 (EL2) and (Tyr185) and formed several H-bond interactions with their sidechains.

Our model also revealed some important differences in the interaction of GPR17 with ligands' adenine moiety. According to previous modeling studies on P2Y_1 _[27,35], basic residues in 3.29, 7.39 and 6.55 coordinated the phosphates of adenine ligands such as MRS2179 and related compounds. Moreover, residue 7.36 was H-bound to N6 and N7, whereas residue 7.43 was H-bound to N1. Finally, highly conserved residues in 1.39 and 2.53 contributed to the stabilization of the adenine ring. In the case of GPR17, the 7.36 residue is an arginine, which seemed involved in a persistent ionic interaction with a glutamic acid in 1.31, an acidic residue which is not present in any other P2Y receptors. This pair is likely to form a bridge between TM7 and TM1 close to the binding cavity, thus stabilizing the helical bundle. In GPR17, another basic residue in 2.60 (Arg87) replaces the 7.36 residue facing the binding pocket which is present in the other P2Y receptors. Arg87 formed a H-bond with the adenine N6 of both ligands and also acted as a H-bond donor for adenine N7 of MRS2179. Interestingly, in the absence of ligands, Arg87 formed a salt bridge with Asp41. This pair belonged to the hydrophilic network between TM1, TM2, TM3 and TM7, suggesting that, in GPR17, the Arg87 residue could play a significant role in ligand-mediated activation mechanism. Also in the case of GPR17, for both MRS2179 and cangrelor, our data highlighted a particularly important role for residue 6.55 in phosphate coordination. For time evolution plots of the main interactions observed between GPR17 and docked ligands during MD see Additional file: Figure 10, Additional file: Figure 11 and Additional file: Figure 12 [see Additional file 1].

The network of H-bonds between GPR17 polar residues of TM1, TM2, TM3, TM7 and water remained stable throughout the MD simulation.

Overall, the main functional regions for receptor binding in UDP, MRS2179 and cangrelor overlapped: atoms N1 in uridine and N9 in adenine were superimposed. The 3'-phosphate group of MRS2179 and the α-phosphate group of UDP and cangrelor overlapped, while the ribose approximately occupied the same spatial regions at the centre of the helical bundle. In summary, for the natural ligand UDP, the uracil ring pointed toward TM1 and TM2, while also binding to TM7, TM3 and TM1. The diphosphate moiety was bound to TM3 and TM6 and pointed toward TM5, TM6 and EL2. For MRS2179, the adenine ring was bound to TM7 and TM3 and pointed toward helices TM1 and TM2. The phosphate moiety was bound to TM3, TM7, TM6 and EL2, with the 5'-phosphate pointing towards EL2 and the 3'-phosphate pointing towards TM5 and TM6. Finally, also for cangrelor, the adenine ring was positioned between TM3 and TM7, while the phosphate chain was comprised between TM3, TM5, TM6 and extended towards EL2.

## Conclusion

The dynamic simulations of a natural purinergic ligand and of two strong purinergic antagonists suggest that the agonist/antagonist binding modes to the new nucleotide receptor GPR17 are comparable, and that the topology of the binding site is the same. The agonistic or antagonistic nature of the different ligands cannot be determined from the analysis of the binding mode, since the binding site itself appears to be shared by both nucleotide agonists and antagonists. The binding region corresponds to the well described nucleotide binding site of the other P2Y receptors, at least for some crucial interactions (e.g., with residue 6.55). Some important differences were, however, noticed, since only one of the three basic residues that are typically present in the binding pocket of P2Y receptors is conserved in GPR17. Thus, the so called "nucleotide binding site" is not defining a single binding mode and surely not a unique configuration of nucleotides bound to it. Recent developments show heterogeneity in the modalities of nucleotide binding also among the already known members of the P2Y receptor family [31]. For example, for P2Y_14_, it has been suggested that two of the basic residues (6.55 and 7.35) that have been previously involved in binding to nucleotide phosphates, are instead bound to the hexose moiety of sugar-nucleotides. Moreover, initial docking and MD experiments suggest that, in a similar way to P2Y_1 _[42], there may be an additional "accessory" binding site also on GPR17, in a region located at the interface between the extracellular environment and the helical bundle. Here, some key aminoacids in EL3 and in Nt could drive the efficacy with which small ligands are guided into the helical bundle, and could consequently affect receptor response. Interestingly, in our previous [^35^S]GTPgammaS binding studies, we noticed a marked species difference in the potency of some purinergic agonists and antagonists between the rat and human receptor [13]. At present, we have no obvious explanation to explain this difference. However, despite the absence of marked sequence changes between the two receptors, there are indeed qualitatively important differences in single aminoacid residues belonging to the EL3 and Nt, in the receptor region highlighted above. Future mutagenesis studies guided by these species differences will help clarifying the relative importance of this receptor region in both ligand binding and receptor activation.

GPR17 has been also reported to bind to cysteinyl-LTs [13]. Our model suggests that neither the dimensions nor the dynamics of the receptor with or without ligands will accommodate the leukotrienic ligands in the nucleotide binding site. Therefore, a first clear hint coming out from this work is that this dual receptor most probably has a dual binding mechanism, according to the chemical entity that is going to bind. Based on our modeling, we hypothesise that leukotrienic ligands may extend well beyond the nucleotide binding pocket, therefore also involving the extracellular loops. This conclusion is consistent with our previous experimental data with [^35^S]GTPgammaS binding [13]. In 1321N1 cells heterologously expressing *h*GPR17, blockade of the cysteinyl-LT binding site with the CysLT antagonists montelukast or pranlukast did not abolish the response to uracil derivatives. In a similar way, blockade of the nucleotide binding site with either cangrelor or MRS2179 still permitted the response to LTD_4_. Moreover, incubation with both LTD_4 _and uracil derivatives, utilized at concentrations corresponding to their EC_50 _values, resulted in an augmented response with respect to responses induced by each agonist alone [13]. Globally, these data suggest that two distinct binding sites, or at least two binding modes (one for nucleotides and the other one for cysteinyl-LTs), are present on GPR17. We envisage that the elucidation of this second binding site for cysteinyl-LTs in GPR17 will help designing novel "hybrid" dual antagonists of previously unexplored therapeutic potential. This could be achieved by the rational design of a dual ligand carrying structural elements from two distinct classes of ligands. This approach would also meet the requirements of the current lead-discovery strategy that aims at the development of compounds that can simultaneously target different biological pathways. This approach can be advantageous in the case of complex mechanisms of action for which the blockade of a single mechanism fails to sort the desired effect [45].

## Methods

### Homology modeling

The aminoacidic sequences of *b*Rh, *h*GPR17 and pharmacologically related P2Y and CysLT receptors were obtained from Swiss-Prot/TrEMBL database at the ExPaSy server [46]. Multiple sequences alignment was performed using the on-line available Clustal W program accessible through the European Bioinformatic Institute [47,48]. For the homology modelling, we selected the sequence of the shortest isoform of *h*GPR17 [GPCRDB: Q13304-2], that is 339 aminoacid-long and lacks the starting Nt 28 residues that are present in the full-length isoform. We chose not to take into account the initial portion of the Nt domain since all the experimental data that are available on GPR17 have been obtained with the pharmacologically active shortest isoform of *h*GPR17 [13]. Moreover, the packing of the receptor was not influenced by this dope. In addition, there are evidences that, in all GPCRs, the Nt domain includes several glycosylation sites, thus making the results of the 3-D modeling even more speculative. The alignment between *h*GPR17 and *b*Rh sequences to the NEST [49] module of the Jackal package 1.5 was imported to obtain an initial approximate 3-D structure of GPR17. The model was built using the X-ray crystallographic coordinates of the *b*Rh structure at 2.2 Å [19], deposited at the RCSB Protein Data Bank [50], as a template [PDB: 1U19]. The crude initial model was topologically close to the template. However, since, with respect to *b*Rh, GPR17 has an additional pair of cysteines which is conserved in all P2Y and CysLT receptors, the existence of an additional extracellular disulphide bridge was assumed, and this presumptive bridge was added via computer graphics, by linking the two conserved Cys23 and Cys269 residues in the Nt and EL3, respectively.

### Molecular dynamics simulations

The 3-D molecule was locally minimized *in vacuo *by constraining the backbone of the helices in order to give a first optimization of the rough geometry derived from homology modeling.

The model of DPPC in the liquid-crystalline phase proposed by Tieleman and Berendsen [51,52] was then used to reproduce the membrane environment. The system consisting of 128 lipids and 3655 water molecules, available on-line and completed with its relative simulation parameters [53], was used as the initial configuration.

The structure of *h*GPR17 was manually inserted in the centre of the 128 lipid bilayer, in such a way that the principal axes of the helical bundle was parallel to the membrane axis (z) and perpendicular to the membrane plane (xy), and the extracellular and intracellular loops were at the lipid interface. Twenty eight lipid molecules overlapping with the receptor were removed and the system was solvated with the spc216 water model [54] provided in the Gromacs package [55,56].

Additional solvent molecules were included into the system at the water positions reported in the interior of the 1U19 crystallographic structure of *b*Rh. Our receptor model was first superimposed to both chain A and chain B, including the already observed 66 water positions; the 36 non-overlapping internal water molecules were kept in their position.

The resulting system consisted of 339 aminoacid residues, 100 DPPC molecules, 6384 water molecules (6348 of which were external and 36 internal), for a total of 27544 atoms in a rectangular box of 61 × 61 × 67 Å.

The final ensemble was submitted to energy minimization cycles, followed by simulated annealing in order to lead the system to a more favourable energetic condition before starting the pure MD simulation.

During all these simulation steps, the backbone of the seven TM helices, the helix 8 domain and the structured EL2 motifs were constrained to maintain the overall arrangement of the helical bundle and the structural conserved organization of EL2.

The protocol by which the assembly was prepared for the MD run was composed of separated cycles as described below. For the earlier minimization steps, the steepest descent algorithm was applied. Only one or two components of the system were allowed to move in each stage, while the remaining components were fixed. The minimization sequence of the various component of the system was the following: first the lipids, then the water, then both lipids and water, and finally the whole system. The latter was further minimized using the conjugate gradient algorithm, and then a first run of 200 ps of MD simulation at 5 K was performed. At the end of this first minimization and relaxation protocol, a simulated annealing procedure was performed as follows. The system was heated from 5 to 500 K in 240 ps, then cooled from 500 to 300 K and reheated to 500 K in 200 ps. The heating-cooling process was repeated six times; after that, the system was brought back to 5 K. The global duration of the simulated annealing protocol was 1660 ps, during which the backbone of all α-helices and EL2 were potentially restrained using a force constant of 1000 (kJmol^-1^nm^-2^). The analysis of the dynamic behaviour of the assembly was performed on the energetically minimized structure obtained above. The system was reheated up to 310 K in 400 ps and kept at this temperature for further 240 ps by using a force constant of 100 (kJmol^-1^nm^-2^). Ten ns of dynamics simulation were then performed without any constraints in order to explore conformational changes of the receptor protein under constant standard conditions. The reliability of the MD simulation methodology was then compared with the results obtained from a "trial" run performed applying NOE distance restraints to the interhelical H-bond distances for 3 ns. In particular, the restraints were applied for the distances between the backbone carbonyl oxygen atom of the residue "n" and the backbone NH-group of the residue "n+4" of TM7, with the exception of the prolines. After the first 3 ns, the restraints were removed and the MD simulation was continued for further 3 ns without any restraints.

### Ligand simulations

The interactions between GPR17 and three nucleotide ligands (UDP, MRS2179 and cangrelor) were investigated by means of MD simulations.

The average structure of the system taken during the time frame from 3 to 6 ns of the MD was used for the ligand-receptor simulations. The three ligands were docked into the spatial region corresponding to the putative nucleotide binding pocket for P2Y receptors. To ensure that the starting ligand/receptor complex configurations were energetically favourable, docking experiments were conducted on the average structure of MD described above. Briefly, the average structure was initially submitted to a binding-cavity search using the Sitefinder tool included in the Delos package [43]. A transmembrane region spatially overlapping to the nucleotide binding pocket suggested for the other nucleotide-activating receptors was found on GPR17. UDP was docked in the pocket with the same orientation expected for nucleotide-receptor complexes: the docked ligand together with the sidechains of the residues within 4.5 Å distance were then locally minimized. Docking experiment were performed using the docking tool for rigid ligands included in the Delos package. The standard simulated-annealing protocol provided in the Delos package was used for the docking protocol. The charge state of the ligands was computed using the converter software VEGA [57] with the standard Gasteiger-Marsili's method [58]. The best energy scoring obtained by UDP and consistent with our previous hypothesis on ligand orientation was used as a starting configuration for the MD run. MRS2179 and cangrelor starting configurations for MD were instead defined via superimposition with the final UDP placement and then re-docked into the putative binding pocket using Delos. Ligand's superimposition and the starting coordinates of the ligands were obtained using Moe [59]. Ligands topology were obtained from the automatic server PRODRG [60,61] using the standard Gromacs forcefield. The ligands were inserted into the binding pocket mentioned before and the systems were prepared for the MD simulations using a stepwise protocol. First, the systems were gradually minimized via the steepest descent allowing the various components to move individually with the following order: solvent and lipids, sidechains, the whole systems. The conjugate gradient method was then applied to improve the energy content of the systems. The three ligand-receptor-membrane complexes were heated to the simulation temperature of 310 K in 300 ps and the three MD runs were started using the general conditions defined for this study and described below. For UDP and MRS2179, 3 ns of MD run were computed; for cangrelor the MD run was extended to 6 ns.

### Computational details

MD simulations were run on a Linux cluster Blade with Xion processors. All minimization steps, MD simulations and analysis were curried out using the Gromacs 3.3 package [55,56]. All the MD simulations were performed using the standard Gromacs force field; the periodic boundary conditions were applied in all three x, y and z dimensions. The isothermal isobaric NPT ensemble (constant number of particles, pressure and temperature) was applied. Each component of the system was separately coupled to a temperature bath at 310 K, with a coupling constant τ_t _of 0.1 ps. The pressure coupling was set as independent in the x and y directions (semi isotropic coupling), with a constant pressure of 1 bar and a coupling constant τ_p _of 1 ps. A 2 fs time step was used for the integration of the equations of motions and all bond distances involving hydrogen atoms were constrained using LINCS [62]. Coulombian interactions were treated with the PME model and a twin range of cut-off radius of 1.8 nm for both the electrostatics and the van Der Waals interactions was used. Configurations were saved for every 1 ps for analysis.

## Authors' contributions

CP carried out the computational study, performed the analysis of the data and drafted the manuscript, GR participated in the drafting and in the design of the study, MPA conceived of the study, provided the biological background for data interpretation and helped to draft, PF participated in its design and coordination. All authors read and approved the final manuscript.

## Supplementary Material

Additional file 1 Additional file Figures content: Multiple alignment of the sequences of *h*GPR17, P2Y receptors, CysLT_1_, CysLT_2 _and *b*Rh; evolution of the energy of the system; rmsd of the C-α of the two compared MD simulations, with or without distance restraints as a function of time; general topology of the receptor protein; superimposition of GPR17 before and after the simulation; number of H-bonds and number of residues with α-helix geometry observed during the two compared MD simulations, with or without distance restraints; time evolution of the interactions occurring between the receptor, internal water molecules and ligands during the simulations of the receptor-membrane system. Click here for file
