# Supplementary material for: GPR17: Molecular modeling and dynamics studies of the 3-D structure and purinergic ligand binding features in comparison with P2Y receptors
Source: BMC Bioinformatics. 2008 Jun 4;9:263. doi: 10.1186/1471-2105-9-263 (PMC2443813; doi:10.1186/1471-2105-9-263)

Additional file Figures

GPR17: Molecular modeling and dynamics studies of the 3-D structure and purinergic ligand binding features in comparison with P2Y receptors

Chiara Parravicini, Graziella Ranghino, Maria P Abbracchio, Piercarlo Fantucci

Table of contents

Additional file: Figure 1...2

Additional file: Figure 2...4

Additional file: Figure 3...5

Additional file: Figure 4...6

Additional file: Figure 5...7

Additional file: Figure 6...8

Additional file: Figure 7...9

Additional file: Figure 8...10

Additional file: Figure 9...11

Additional file: Figure 10...13

Additional file: Figure 11...14

Additional file: Figure 12...15

Additional file: Figure 1. Multiple alignment of the sequences of bovine rhodopsin (*b*Rh), P2Y, CysLT$_1$, CysLT$_2$ receptors and *h*GPR17. The cysteines involved in the formation of the two assumed disulphide bridges are highlighted in red; the H-X-X-R/K motif typical of all the P2Y and CysLTs receptor subtypes and conserved in GPR17 is highlighted in green.

```
                 NT                 TM1                             IL1
bRh        26 APQYYLAEP---------WQFSMLAAYMFLLIMLGFPINFLTLYVTVQHK--K-LRTP
P2Y1       40 FKCALTKTG---------FQFYYLPAVYILVFIIGFLGNSVAIWMFVFHM--K-PWSG
P2Y2       23 YRCRF-NED---------FKYVLLPVSYGVVCVLGLCLNAVALYIFLCRL--K-TWNA
P2Y4       25 LDCWF-DED---------FKFILLPVSYAVVFVLGLGLNAPTLWLFIFRL--R-PWDA
P2Y6       16 TTCVY-REN---------FKQLLLPPVYSAVLAAGLPLNICVITQICTSR--R-ALTR
P2Y11      17 KSCPANFLAAADDKLSG-FQGDFLWPILVVEFLVAVASNGLALYRFSIRKQ-R-PWHP
P2Y12      15 SLCTR-DYK---------ITQVLFPLLYTVLFFVGLITNGLAMRIFFQIR----SKSN
P2Y13      13 ERCPR-DTR---------IVQLVFPALYTVVFLTGILLNTLALWVFVHIP----SSST
P2Y14      12 ESCSQ-NLL---------ITQQIIPVLYCMVFIAGILLNGVSGWIFFYVP----SSKS
CysLT1     12 ATCHDTIDD---------FRNQVYSTLYSMISVVGFFGNGFVLYVLIKTY--H-KKSA
CysLT2     29 RNCTIEN-----------FKREFFPIVYLIIFFWGVLGNGLSIYVFLQPY--K-KSTS
GPR17      21 EQCGQ-ETP---------LENMLFASFYLLDFILALVGNTLALWLFIRDH--K-SGTP
```

```
            TM2                                EL1     TM3
bRh        LNYILLNLAVADLFMVFGGFTTTLYTSLHGYFVFGPTGCNLEGFFATLGGEIALWSLVVL
P2Y1       ISVYMFNLALADFLYVLTLPALIFYYFNKTDWIFGDAMCKLQRFIFHVNLYGSILFLTCI
P2Y2       STTYMFHLAVSDALYAASLPLLVYYYARGDHWPFSTVLCKLVRFLFYTNLYCSILFLTCI
P2Y4       TATYMFHLALSDTLYVLSLPTLIYYYAAHNHWPFGTEICKFVRFLFYWNLYCSVLFLTCI
P2Y6       TAVYTLNLALADLLYACSLPLLIYNYAQGDHWPFGDFACRLVRFLFYANLHGSILFLTCI
P2Y11      AVVFSVQLAVSDLLCALTLPPLAAYLYPPKHWRYGEAACRLERFLFTCNLLGSVIFITCI
P2Y12      FIIFLKNTVISDLLMILTFPFKILSDAKLGTGPLRTFVCQVTSVIFYFTMYISISFLGLI
P2Y13      FIIYLKNTLVADLIMTLMLPFKILSDSHLAPWQLRAFVCRFSSVIFYETMYVGIVLLGLI
P2Y14      FIIYLKNIVIADFVMSLTFPFKILGDSGLGPWQLNVFVCRVSAVLFYVNMYVSIVFFGLI
CysLT1     FQVYMINLAVADLLCVCTLPLRVVYYVHKGIWLFGDFLCRLSTYALYVNLYCSIFFMTAM
CysLT2     VNVFMLNLAISDLLFISTLPFRADYYLRGSNWIFGDLACRIMSYSLYVNMYSSIYFLTVL
GPR17      ANVFLMHLAVADLSCVLVLPTRLVYHFSGNHWPFGEIACRLTGFLFYLNMYASIYFLTCI
```

```
                IL2                   TM4                    EL2
bRh        AIERYVVVCKPMSN----FRFGENHAIMGVAFTWVMALACAAPPLVGWSRY-----IPEG
P2Y1       SAHRYSGVVYPLKS---LGRLKKKNAICISVLVWLIVVVAISPILFYSGTG-----VRKN
P2Y2       SVHRCLGVLRPLRS---LRWGRARYARRVAGAVWVLVLACQAPVLYFVTTS-----ARGG
P2Y4       SVHRYLGICHPLRA---LRWGRPRLAGLLCLAVWLVVAGCLVPNLFFVTTS-----NKGT
P2Y6       SFQRYLGICHPLAP--WHKRGGRRAAWLVCVAVWLAVTTQCLPTAIFAATG-----IQRN
P2Y11      SLNRYLGIVHPFFA---RSHLRPKHAWAVSAAGWVLAALLAMPTLSFSHLK-----RPQQ
P2Y12      TIDRYQKTTRPFKT---SNPKNLLGAKILSVVIWAFMFLLSLPNMILTNRQ-----PRDK
P2Y13      AFDRFLKIIRPLRN---IFLKKPVFAKTVSIFIWFFLFFISLPNMILSNKE-----ATPS
P2Y14      SFDRYYKIVKPLWT---SFIQSVSYSKLLSVIVWMLMLLLAVPNIILTNQS-----VREV
CysLT1     SFFRCIAIVFPVQN---INLVTQKKARFVCVGIWIFVILTSSPFLMAKPQK-----DEKN
CysLT2     SVVRFLAMVHPFRL---LHVTSIRSAWILCGIIWILIMASSIMLLDSGSE------QNGS
GPR17      SADRFLAIVHPVKS---LKLRRPLYAHLACAFLWVVVAVAMAPLLVSPQTV-----QTNH
```

```
                                            TM5
bRh        MQCSCGIDYYTP---HEE-----------TNNESFVIYMFVVHFIIPLIVIFFCYGQL-V
P2Y1       KTITCYDT--TS---DEY----------LRSYFIYSMCTTVAMFCVPLVLILGCYGLI-V
P2Y2       -RVTCHDT--SA---PEL----------FSRFVAYSSVMLGLLFAVPFAVILVCYVLM-A
P2Y4       -TVLCHDT--TR---PEE----------FDHYVHFSSAVMGLLFGVPCLVTLVCYGLM-A
P2Y6       -RTVCYDL--SP---PAL----------ATHYMPYGMALTVIGFLLPFAALLACYCLL-A
P2Y11      GAGNCSVA--RP---EACIKCLGTADHGLAAYRAYSLVLAGLGCGLPLLLTLAAYGAL-G
P2Y12      NVKKCSFL--KS---EFG-----------LVWHEIVNYICQVIFWINFLIVIVCYTLI-T
P2Y13      SVKKCASL--KG---PLG-----------LKWHQMVNNICQFIFWTVFILMLVFYVVI-A
P2Y14      TQIKCIEL--KS---ELG-----------RKWHKASNYIFVAIFWIVFLLLIVFYTAI-T
CysLT1     -NTKCFEP--PQ--DNQT----------KNHVLVLHYVSLFVGFIIPFVIIIVCYTMI-I
CysLT2     -VTSCLEL--NL---YK-----------IAKLQTMNYIALVVGCLLPFFTLSICYLLI-I
GPR17      -TVVCLQL--YR---EK--------------ASHHALVSLAVAFTFPFITTVTCYLLI-I
```

```
              IL3                   TM6                            EL3
bRh           FTVKEAAAQQQES--ATTQKAEKEVTRMVIIMVIAFLICWLPYAGVAFYIFTHQG-----
P2Y1          RALIYKDLD--------NSPLRRKSIYLVIIVLTVFAVSYIPFHVMKTMNLRARLDF--Q
P2Y2          RRLLKPAYGTSG----GLPRAKRKSVRTIAVVLAVFALCFLPFHVTRTLYYSFRS-----
P2Y4          RRLYQPLPGSA------QSSSRLRSLRTIAVVLTVFAVCFVPFHITRTIYYLARL-----
P2Y6          CRLCRQDGPAEP----VAQERRGKAARMAVVVAAAFAISFLPFHITKTAYLAVRS----T
P2Y11         RAVLRSPGM--------TVAEKLRVAALVASGVALYASSYVPYHIMRVLNVDARR----R
P2Y12         KELYRSYVRTRG----VGKVPRKKVNVKVFIIIAVFFICFVPFHFARIPYTLSQT----R
P2Y13         KKVYDSYRKSKS----KDRKNNKKLEGKVFVVVAVFFVCFAPFHFARVPYTHSQT----N
P2Y14         KKIFKSHLKSSR----NSTSVKKKSSRNIFSIVFVFFVCFVPYHIARIPYTKSQT----E
CysLT1        LTLLKKSMKK-------NLSSHKKAIGMIMVVTAAFLVSFMPYHIQRTIHLHFLH----N
CysLT2        RVLLKVEVPESG-----LRVSHRKALTTIIITLIIFFLCFLPYHTLRTVHLTTWK-----
GPR17         RSLRQGLRV--------EKRLKTKAVRMIAIVLAIFLVCFVPYHVNRSVYVLHYR----S

                         TM7                                CT
bRh           ------------------SDFGP----IFMTIPAFFAKTSAVYNPVIYIMMN------------
P2Y1          TPAMC-------AFNDRVY----ATYQVTRGLASLNSCVDPILYFLAG------------
P2Y2          LDLSC-------HTLNAIN----MAYKVTRPLASANSCLDPVLYFLAGQRLVRFARDAKP
P2Y4          LEADC-------RVLNIVN----VVYKVTRPLASANSCLDPVLYLLTG------------
P2Y6          PGVPC-------TVLEAFA----AAYKGTRPFASANSVLDPILFYFTQ------------
P2Y11         WSTRCPSFADIAQATAALELGPYVGYQVMRGLMPLAFCVHPLLYMAAV------------
P2Y12         DVFDC-------TAENTLF----YVKESTLWLTSLNACLDPFIYFFLC------------
P2Y13         NKTDC-------RLQNQLF----IAKETTLFLAATNICMDPLIYIFLC------------
P2Y14         AHYSC-------QSKEILR----YMKEFTLLLSAANVCLDPIIYFFLC------------
CysLT1        ETKPC-------DSVLRMQ----KSVVITLSLAASNCCFDPLLYFFSG------------
CysLT2        -VGLC---------KDRLH----KALVITLALAAANACFNPLLYYFAG------------
GPR17         HGASC-------ATQRILA----LANRITSCLTSLNGALDPIMYFFVA------------

bRh           -------KQFRNCMVTTLCCGKNP 327
P2Y1          -------DTFRRRLSRATRKASRR 345
P2Y2          PTGPSPATPARRRLGLRRSDRTDM 346
P2Y4          -------DKYRRQLRQLCGGGKPQ 327
P2Y6          -------KKFRRRPHELLQKLTAK 322
P2Y11         -------PSLGCCCRHCPGYRDSW 342
P2Y12         -------KSFRNSLISMLKCPNSA 319
P2Y13         -------KKFTEKLPCMQGRKTTA 317
P2Y14         -------QPFREILCKKLHIPLKA 316
CysLT1        -------GNFRKRLSTFRKHSLSS 316
CysLT2        -------ENFKDRLKSALRKGHPQ 326
GPR17         -------EKFRHALCNLLCGKRLK 318
```

Additional file: Figure 2. Total energy as a function of time of the entire membrane-receptor system during 10 ns of MD simulation. After an initial decrease, the total energy of the system kept a stable trend at least for the lasts 3 ns of simulation, indicating that an equilibrium state had been reached.

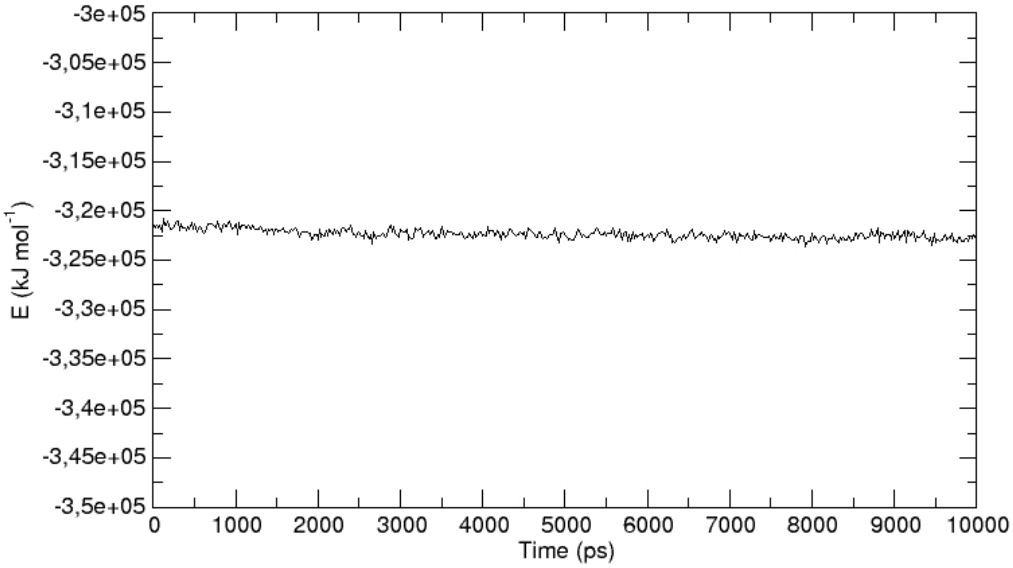

Additional file: Figure 3. Rmsd of the C-α atoms of the whole protein during the MD simulations performed with or without NOE distance restraints. The plot shows the rmsd of C-α atoms computed for the two types of simulations performed with NOE distance restraints (Condition 1, 6 ns, black) or without NOE distance restraints (Condition 2, 10 ns, red) (see Methods for more details). After an initial increase, the rmsd values remained stable with time and always below 1.5 Å, indicating that the overall structure of the receptor remained stable and comparable during the course of both types of simulations.

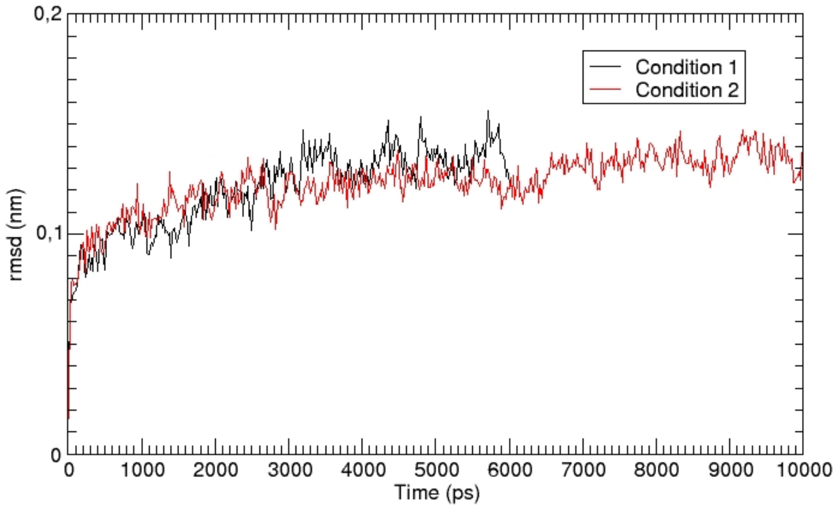

Additional file: Figure 4. General topology of the refined model of GPR17. Representative snapshot of the MD trajectory obtained after 10 ns of simulation. Typical secondary structures of the receptor are highlighted: α -helices are in orange, β -sheets in yellow, turns in green and blue, and loops in turquoise. The nine internal water molecules are represented in red/white as spheres.

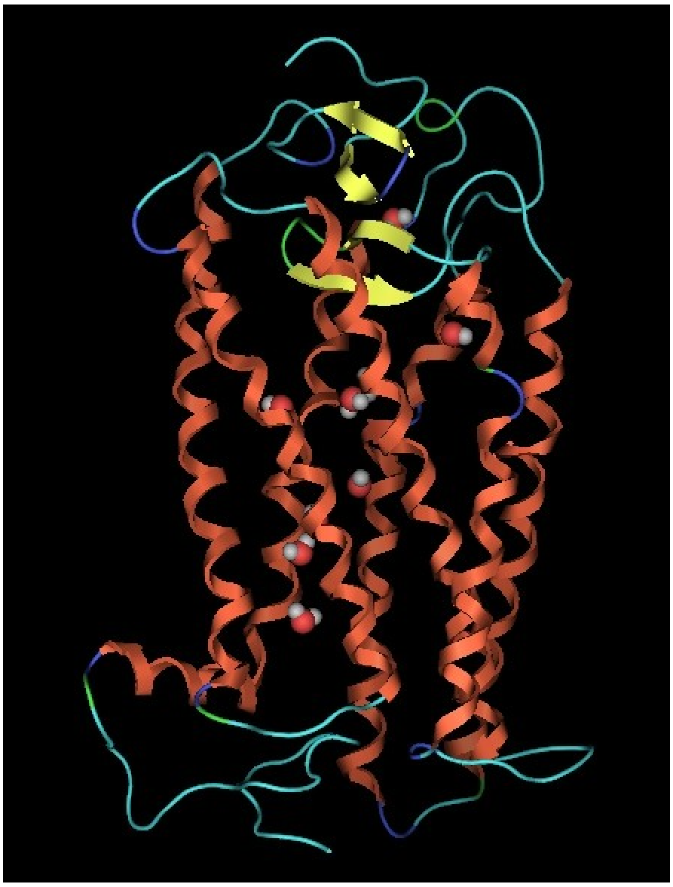

Additional file: Figure 5. Superposition of the GPR17 models before (in red) and after (in blue) the simulation in lipide-water environment. With the exception of TM7, which showed a higher mobility with respect to the other TM domains, the arrangement of α -helices underwent little changes during the MD simulation. Among the loop regions, IL1, IL3 and EL1 were the most rigid, whereas EL2 moved towards the transmembrane domain and displayed a new network of contacts. As expected, the Nt and Ct regions were by far the most mobile regions.

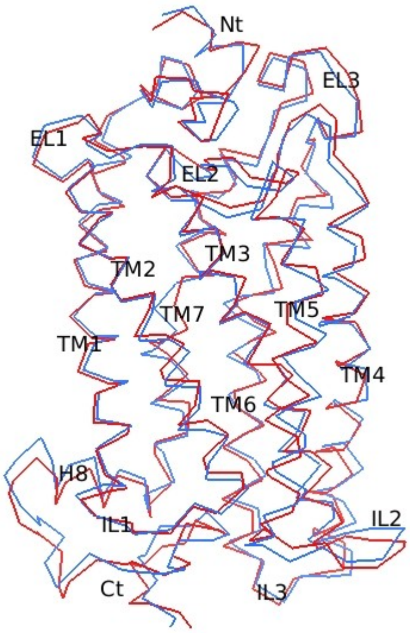

Additional file: Figure 6. Number of the H-bonds observed during the MD simulations performed with or without NOE distance restraints. The plots show the number of H-bonds observed during the two types of simulations performed with NOE distance restraints (Condition 1, 6 ns) or without NOE distance restraints (Condition 2, 10 ns) (see Methods for more details). Panel a shows the number of H-bonds computed for the whole receptor protein (yellow and brown lines for Conditions 1 and 2, respectively) and for the receptor backbone (blue and green lines for Conditions 1 and 2, respectively). Panel b shows the H-bond number fluctuations for TM7 only (black and red lines for Conditions 1 and 2, respectively). In all cases, the number of H-bonds was comparable for the two simulations, suggesting a similar dynamic behaviour and stability.

a

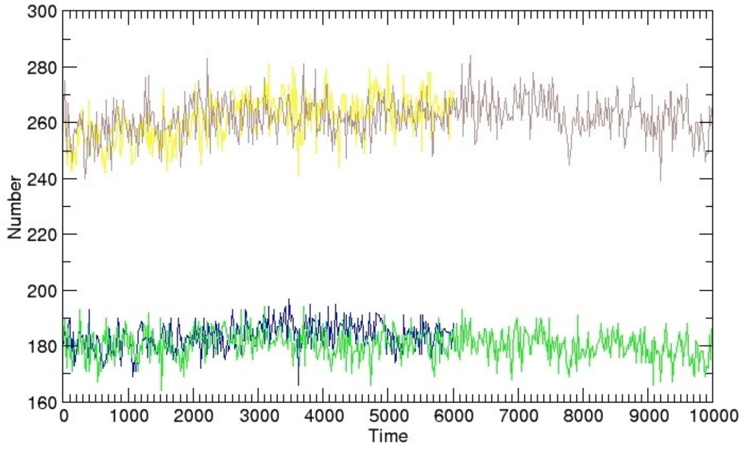

b

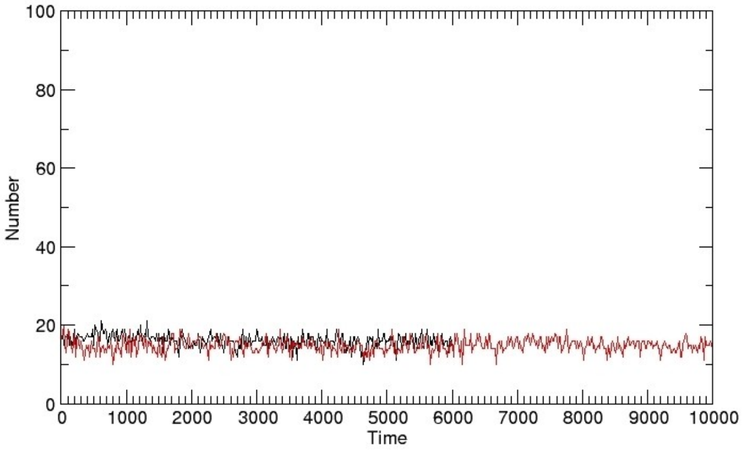

Additional file: Figure 7. Number of residues with α -helix conformation observed during the MD simulations performed with or without NOE distance restraints. The plot shows the number of residues with α -helix conformation observed during the two types of simulations performed with NOE distance restraints (Condition 1, 6 ns, in black) or without NOE distance restraints (Condition 2, 10 ns, in red) (see Methods for more details). The number of residues showing an α -helix conformation is very similar in both cases, suggesting that no significant differences in terms of stability and structure of helices arise from the two MD simulation methods.

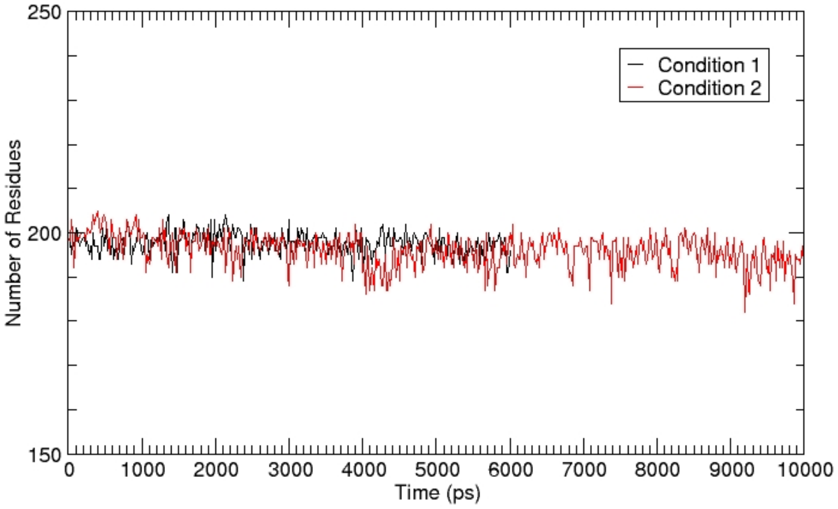

Additional file: Figure 8. Time evolution of principal H-bonds formed by internal water molecules during the 10 ns of MD simulation of the receptor-membrane system. Plots show the distances between H-bond donor/acceptor groups of selected internal water molecules and sidechains that formed multiple H-bond interactions during the simulations. Panel a shows the network involving Tyr297, Asn49 and two molecules of water (Wat6809, Wat6816); panel b shows the interaction between Arg87 and Asp77, mediated by two molecule of internal water (Wat6815, Wat6817), and the interaction between Asp41 and water Wat6817. In addition, Arg87 engaged a stable direct electrostatic interaction with Asp41 as shown in Additional file: Figure 9 b.

a

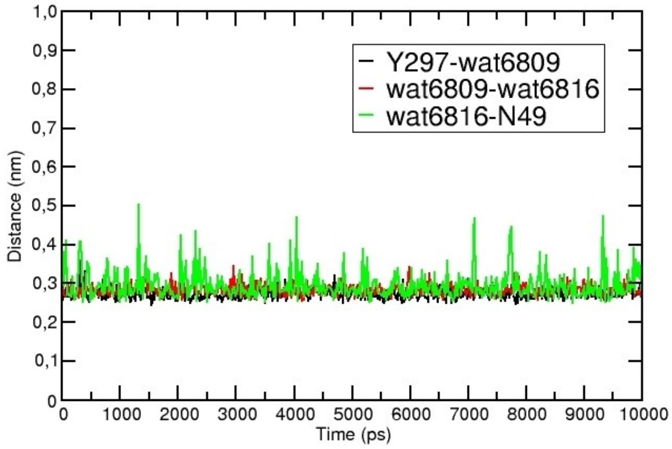

b

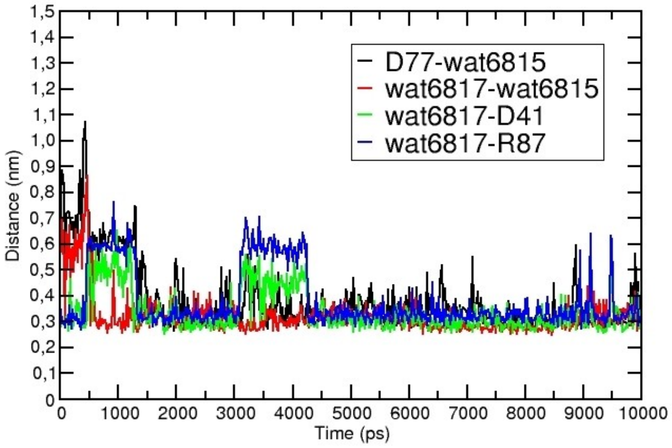

Additional file: Figure 9. Time evolution of selected distances obtained from 10 ns of simulation of the receptor-membrane system. Plots show the distances between donor/acceptor groups of residues involved in H-bonds or electrostatic interactions. Panel a plots the H-bonds formed during the MD between Asp77 and the three surrounding asparagine residue: Asn49 (in black), Asn114 (in red) and Asn289 (in green). This multiple interaction defined a network between TM1, TM2, TM3 and TM7. Panel b shows the H-bond observed between Ser118 (TM3) and Asp293 (TM7) (in green). Distance between the guanidine group of Arg87 and carboxyl Asp41 is plotted in black: the two groups carrying opposite charges formed a salt bridge during the MD run. Panel c shows H-bonds formed by residues belonging to EL2 with helices. The sidechain of Tyr185 pointed toward the transmembrane space and engaged a stable interaction with Tyr112 (TM3) (in black); Gln183 oriented its sidechain inside the helical bundle and could alternatively interact with both Tyr116 (TM3) and Ser196 (TM5). This double chance of Gln183 could explain the high level of fluctuation of the observed H-bond lengths for both Gln183-Tyr116 (in red) and Gln183-Ser196 (in blue).

a

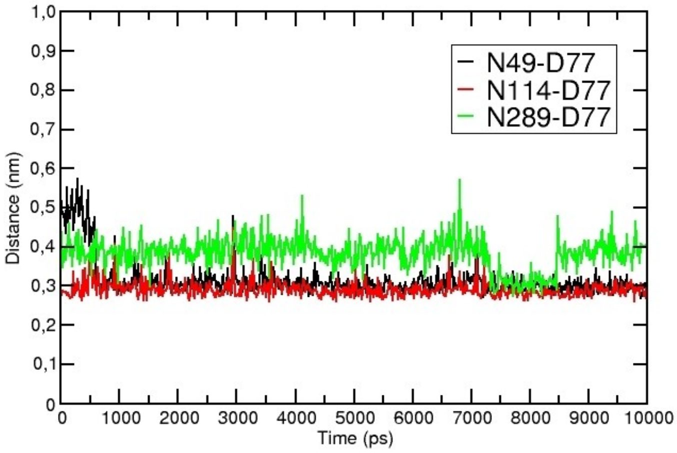
N49-D77
N114-D77
N289-D77
Distance (nm)
Time (ps)
1,0
0,9
0,8
0,7
0,6
0,5
0,4
0,3
0,2
0,1
0
0
1000
2000
3000
4000
5000
6000
7000
8000
9000
10000

b

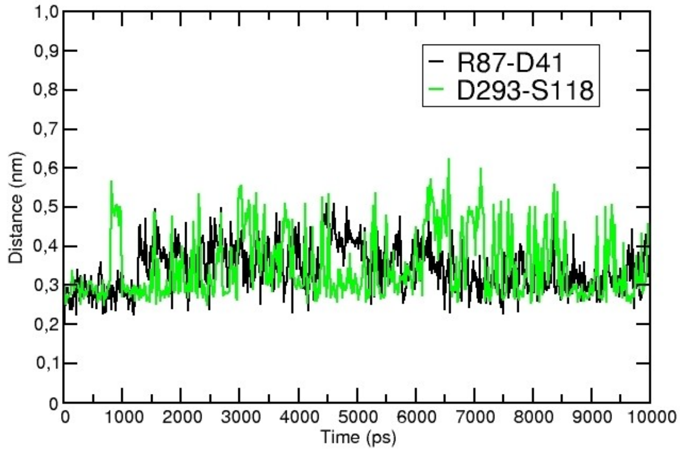
R87-D41
D293-S118
Distance (nm)
Time (ps)
1,0
0,9
0,8
0,7
0,6
0,5
0,4
0,3
0,2
0,1
0
0
1000
2000
3000
4000
5000
6000
7000
8000
9000
10000

c

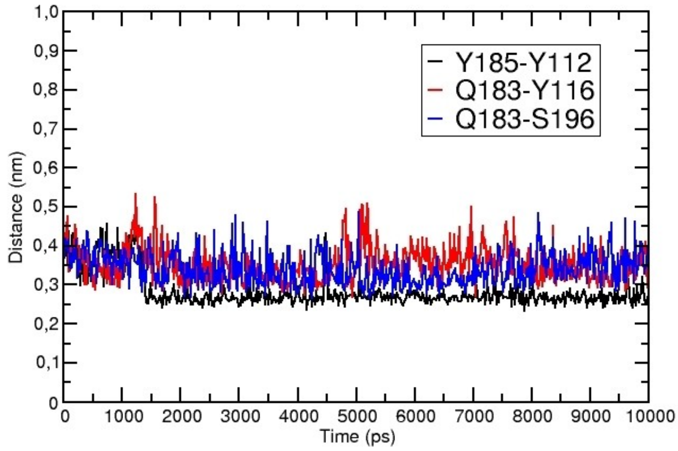
Y185-Y112
Q183-Y116
Q183-S196
Distance (nm)
Time (ps)
1,0
0,9
0,8
0,7
0,6
0,5
0,4
0,3
0,2
0,1
0
0
1000
2000
3000
4000
5000
6000
7000
8000
9000
10000

Additional file: Figure 10. Time evolution of the main interactions occurring between GPR17 and UDP during 3 ns of MD simulation. Plots show the distances between donor/acceptor groups of atoms involved in H-bonds or electrostatic interactions within the ligand-receptor complex. Panel a plots the distances between the guanidine group of Arg255 (TM6) and both the α and β phosphates (in black and red, respectively). Distances were between 4 and 5 Å, indicating the electrostatic interaction between Arg255 and the phosphate moiety as the main driving force for ligand binding. In Panel b other possible interactions between the phosphate chain and residues belonging to the binding pocket are reported. The sidechains of Tyr185 (EL2), Tyr112 (TM5), His252 (TM6) and Tyr262 (TM6) were likely to engage H-bonds with the α -phosphate, while, during the MD run, Gln183 pointed its sidechain to the phosphate chain. Panel c plots the principal H-bonds observed between the uridine moiety and GPR17: interaction engaged by the 4-O group of uracil ring with Tyr38 (TM2) is reported in black, while its interaction with Ser283 (TM7) is reported in red.

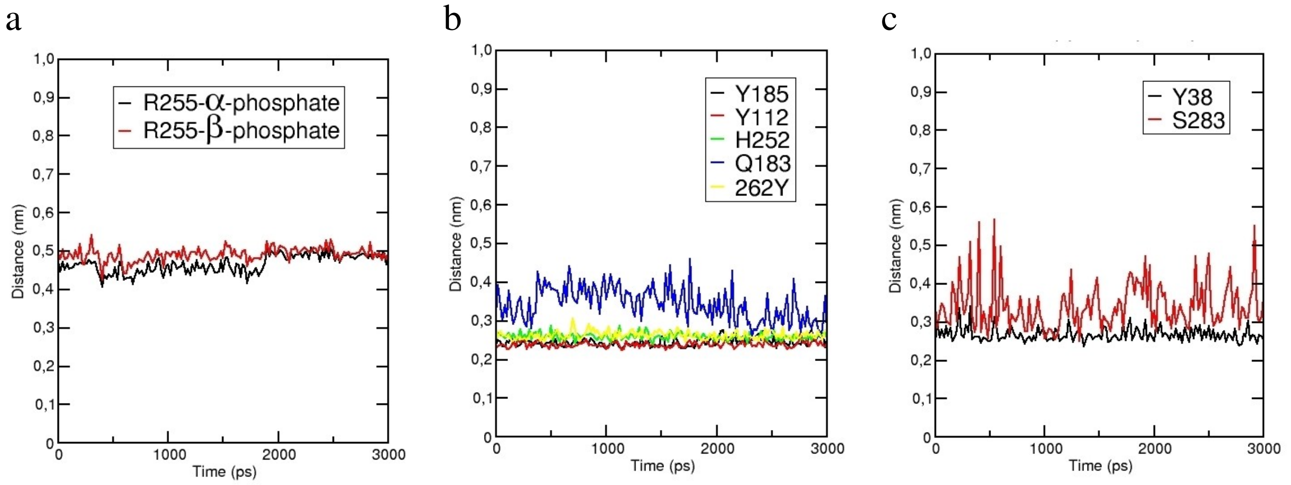

Additional file: Figure 11. Time evolution of the main interactions occurring between GPR17 and MRS2179 during 3 ns of MD simulation. Plots show the distances between donor/acceptor groups of atoms involved in H-bonds or electrostatic interactions within the ligand-receptor complex. Panel a plots the distances between Arg255 (TM6) and both the 3' and 5' phosphates (in black and red, respectively). In analogy with the agonist binding mode the trend of these distances suggests the formation of electrostatic interactions between the oppositely charged phosphate and guanidine groups. Panel b shows the possible H-bonds engaged by the phosphate groups during the simulation: Tyr116 (TM3) and His252 (TM6) formed stable H-bond interactions with the 3'-phosphate (in red and blue, respectively). In the receptor-antagonist complex the tendency of Gln183 (EL2) to point toward TM helices were even more pronounced with respect to the agonist-receptor complex (Additional file: Figure 10) Here, Gln183 could form a H-bond with the 3'-phosphate as showed by the green plot. Tyr112 shifted its sidechain away from the binding cluster, thus permitting the accommodation of the 3'-phosphate (black plot). In Panel c, the distances between adenine moiety and facing residues of the binding pocket are reported: both the N6 and the N7 of the adenine ring could interact with Arg87 (TM2) via H-bond (in red and black, respectively), while the N1 of the adenine ring could interact with Asn114 (TM3) (in green).

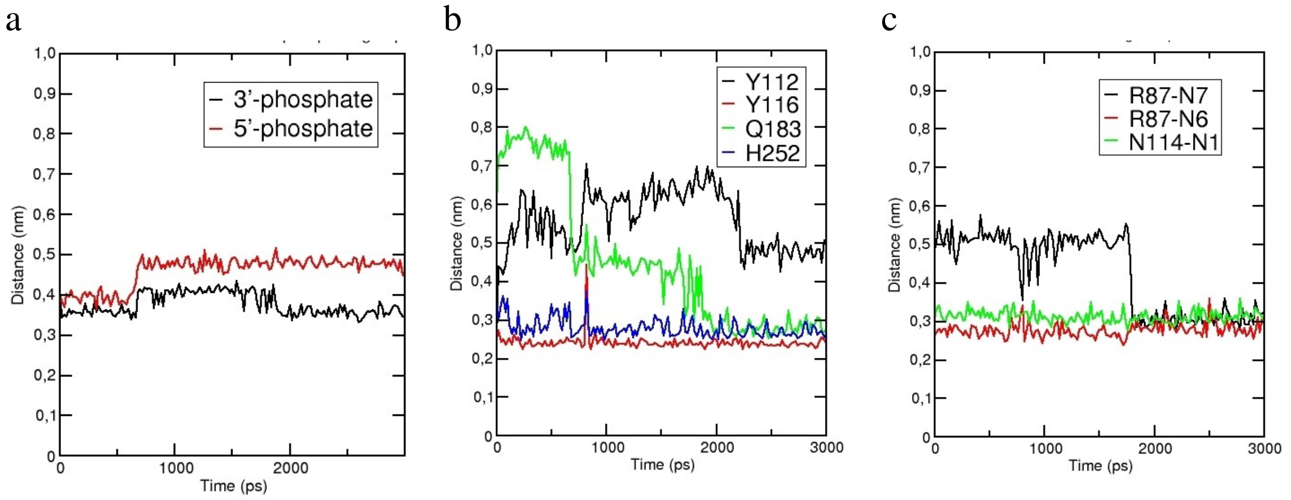

Additional file: Figure 12. Time evolution of the main interactions occurring between GPR17 and cangrelor during 6 ns of MD simulation. Plots show the distances between donor/acceptor groups of atoms involved in H-bonds or electrostatic interactions within the ligand-receptor complex. Panels a, b and c plot the distances between the residues in the binding pocket and the α , β and γ phosphate groups of cangrelor, respectively. In analogy with UDP and MRS2179, the main interactions involved the oppositely charged phosphate (mainly the α –phosphate) and the basic residues Arg255. In addition also Arg186 (EL2) formed electrostatic interaction with the γ -phosphate of cangrelor. In Panel d, distances between the adenine moiety and the facing residues of the binding pocket are reported: as for MRS2179, the N6 of the adenine ring could interact with Arg87 (TM2) via H-bond (in green). The same applies to the N1 of the adenine ring (in red), while the N7 interacted with Gln183 (EL2) (in grey).

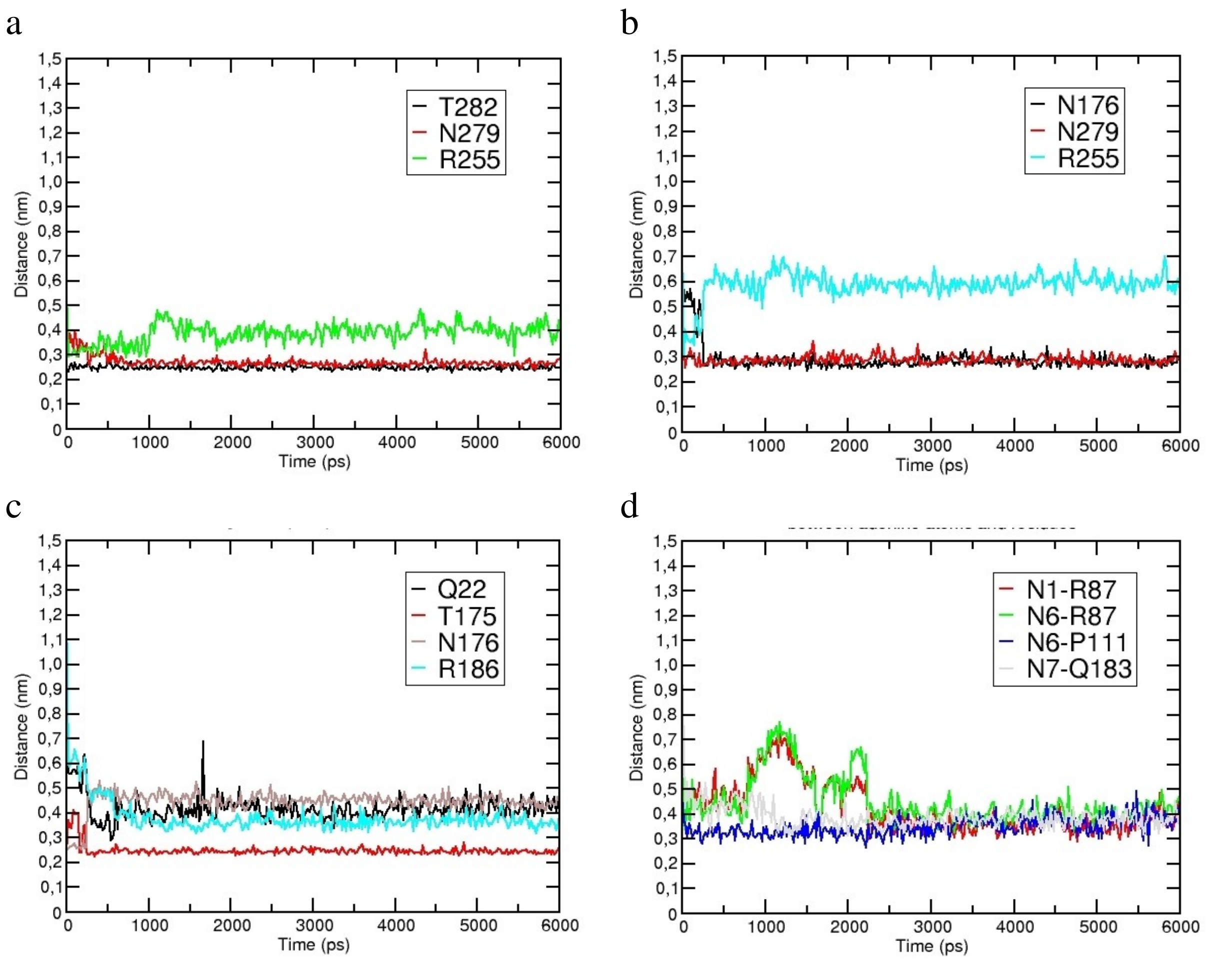

Supplement: Additional file 1 — Additional file Figures content: Multiple alignment of the sequences of hGPR17, P2Y receptors, CysLT1, CysLT2 and bRh; evolution of the energy of the system; rmsd of the C-α of the two compared MD simulations, with or without distance restraints as a function of time; general topology of the receptor protein; superimposition of GPR17 before and after the simulation; number of H-bonds and number of residues with α-helix geometry observed during the two compared MD simulations, with or without distance restraints; time evolution of the interactions occurring between the receptor, internal water molecules and ligands during the simulations of the receptor-membrane system. [file 1471-2105-9-263-S1.pdf]
